# A blood glucose fluctuation-responsive delivery system promotes bone regeneration and the repair function of Smpd3-reprogrammed BMSC-derived exosomes

**DOI:** 10.1038/s41368-024-00328-6

**Published:** 2024-12-01

**Authors:** Lingxiao Wang, Haoqing Yang, Chen Zhang, Yue Zhang, Yilin He, Yang Liu, Pan Ma, Jun Li, Zhipeng Fan

**Affiliations:** 1 Laboratory of Molecular Signaling and Stem Cells Therapy, Beijing Key Laboratory for Tooth Regeneration and Function Reconstruction of Oral Tissues, School of Stomatology, Beijing Stomatological Hospital, Capital Medical University, Beijing, China; 2 Department of Periodontics, School of Stomatology, Beijing Stomatological Hospital, Capital Medical University, Beijing, China; 3 Department of Dental Implant Center, School of Stomatology, Beijing Stomatological Hospital, Capital Medical University, Beijing, China; 4 Beijing Laboratory of Oral Health, Capital Medical University, Beijing, China; 5 Research Unit of Tooth Development and Regeneration, Chinese Academy of Medical Sciences, Beijing, China

**Keywords:** Oral diseases, Regeneration

## Abstract

Blood glucose fluctuation leads to poor bone defect repair in patients with type 2 diabetes (T2DM). Strategies to safely and efficiently improve the bone regeneration disorder caused by blood glucose fluctuation are still a challenge. Neutral sphingophospholipase 2 (Smpd3) is downregulated in jawbone-derived bone marrow mesenchymal stem cells (BMSCs) from T2DM patients. Here, we investigated the effect of Smpd3 on the osteogenic differentiation of BMSCs and utilized exosomes from stem cells overexpressing Smpd3 as the main treatment based on the glucose responsiveness of phenylboronic acid-based polyvinyl alcohol crosslinkers and the protease degradability of gelatin nanoparticles. The combined loading of Smpd3-overexpressing stem cell-derived exosomes (Exos-Smpd3) and nanosilver ions (Ns) to construct a hydrogel delivery system (Exos-Smpd3@Ns) promoted osteogenesis and differentiation of BMSCs in a glucose-fluctuating environment, ectopic osteogenesis of BMSCs in a glucose-fluctuating environment and jawbone regeneration of diabetic dogs in vitro. Mechanistically, Smpd3 promoted the osteogenesis and differentiation of jawbone-derived BMSCs by activating autophagy in the jawbone and inhibiting macrophage polarization and oxidative stress caused by blood glucose fluctuations. These results reveal the role and mechanism of Smpd3 and the Smpd3 overexpression exosome delivery system in promoting BMSC function and bone regeneration under blood glucose fluctuations, providing a theoretical basis and candidate methods for the treatment of bone defects in T2DM patients.

## Introduction

Bone defect diseases have a major impact on human physiological function and mental health.^1–3^ Type 2 diabetes mellitus (T2DM) is a systemic metabolic disease characterized by elevated blood glucose levels that inhibits bone regeneration and increases the risk of bone defects.^4–6^ At present, the treatment methods for bone regenerative disorders caused by T2DM include strict control of blood sugar, application of bone regenerative repair materials, and perioperative infection control. Although these strategies have been implemented, their safety and effectiveness are still insufficient. Therefore, a novel and effective treatment method to promote bone regeneration and repair in T2DM is urgently needed.^7^

Due to ineffective treatment and dietary habits, blood glucose fluctuation is a common phenomenon in T2DM patients during glucose control therapy but causes serious harm to bone tissue regeneration.^8–10^ Fluctuations in blood sugar lead to local microvascular constriction and damage in bone tissue, affecting blood supply and oxygen transport and leading to dysfunction of mitochondrial energy metabolism, increasing oxidative stress levels, and promoting the production of reactive oxygen species (ROS) and matrix metalloproteinases (MMPs), thereby exacerbating local inflammatory reactions. Furthermore, these changes can cause dysfunction of key cells in bone tissue regeneration, such as bone marrow mesenchymal stem cells (BMSCs) and macrophages, leading to impaired bone healing.^8,11–13^ Therefore, strategies to achieve bone regeneration by regulating BMSCs and macrophage function under blood glucose fluctuation conditions are challenging.

With continuous in-depth research on stem cell therapy, researchers have found that the repair of bone tissue by stem cells cannot be achieved without the auxiliary effect of their secreted exosomes in the release of growth factors and signal transmission.^14^ Exosomes are membranous vesicles secreted by cells into the extracellular space, with a diameter of 30–150 nm. These vesicles carry genetic material, lipids, and proteins from the parental cell and have specific physiological functions of the parental cell. Given the nanovesicle properties and physiological functions of exosomes, compared to traditional “foreign” nanocarriers, stem cell exosomes can serve as a natural new type of nanocarrier with anti-inflammatory targeting, low immunogenicity, good stability and biocompatibility. In addition, the nanoscale size and double-layer membrane structure of exosomes give them the stability of colloidal materials, and these structures are not affected by inflammation and free radicals in a damaged environment and can stably exert their anti-inflammatory and repair effects, avoiding issues such as limited lifespan and the need for transportation preservation in stem cell transplantation therapy.^15,16^ However, the application of exosomes mainly faces the following problems: on the one hand, the regenerative ability of exosomes is easily affected by the source of stem cells and cell state, and further modification is usually needed to control the function and state of exosomes by modifying donor cells; this process is relatively simple and will not have adverse effects on the structural integrity of exosomes, which helps to maintain their function;^17,18^ on the other hand, due to the complex pathological environment, such as microvascular circulatory disorders caused by high sugar, high levels of MMPs and free radicals accumulate in the lesion, disrupting the integrity and stability of extracellular vesicle membrane proteins. Therefore, a good carrier is needed to achieve effective delivery of extracellular vesicles.^19^

Neutral sphingophospholipase 2 (Smpd3) is a lipid metabolic enzyme present in bones and cartilage. Smpd3 cleaves myelin and produces ceramides. This molecule is an important intermediate in many metabolic pathways and plays an important role in bone growth and development. In addition, as an important regulator of myelin metabolism in bones, Smpd3 deficiency can damage the mineralization of cartilage and bone extracellular matrix, leading to severe skeletal deformities. Smpd3 knockout mice exhibited severe osteogenesis and developmental impairment, manifested as bone and tooth mineralization disorders, and Smpd3 significantly promoted osteogenic differentiation of periodontal ligament stem cells.^20,21^ In addition, our research group conducted preliminary work on primary cultures of jaw-derived BMSCs from T2DM patients. Through proteomics, we found that Smpd3 may be expressed at low levels in jaw-derived BMSCs from individuals with T2DM, and combined with animal experiments, these data showed that Smpd3 is also expressed at low levels in the bone healing tissue of GK rats.^22,23^ These findings indicate that Smpd3, as an endogenous protein, has good potential in modifying stem cell exosomes to promote blood glucose fluctuations in bone regeneration, but its function and regulatory mechanism are still unclear.

Because of the similarity with those of the natural extracellular matrix, the plasticity and injectability of hydrogels in different bone defects have attracted much attention from scholars, especially the glucose responsiveness of phenylborate-based polyvinyl alcohol crosslinker (TSPBV-PVA) and the degradability of matrix metalloenzymes of gelatin nanospheres, which show good potential as carriers of exosomes to mediate T2DM bone tissue regeneration.^24^ In this study, we utilized TSPBV-PVA and gelatin nanospheres as therapeutic carrier switches, loaded Smpd3-overexpressing BMSC exosomes and nanosilver ions (Ns) and constructed a biomimetic nanodelivery system with the endogenous bone regenerative protein Smpd3 that is responsive to blood glucose fluctuation (Exos-Smpd3@Ns). We evaluated Smpd3’s bone regenerative mechanism, heterotopic osteogenic effect on blood glucose fluctuation, anti-inflammatory effect and jawbone repair effect in diabetic dogs to explore the precise regulation of the T2DM bone defect repair process and provide a theoretical basis and candidate methods for clinical trials to treat T2DM patients with bone defects.

## Results

### Smpd3 promotes osteogenic differentiation of jawbone-derived BMSCs

**Fig. 1:**
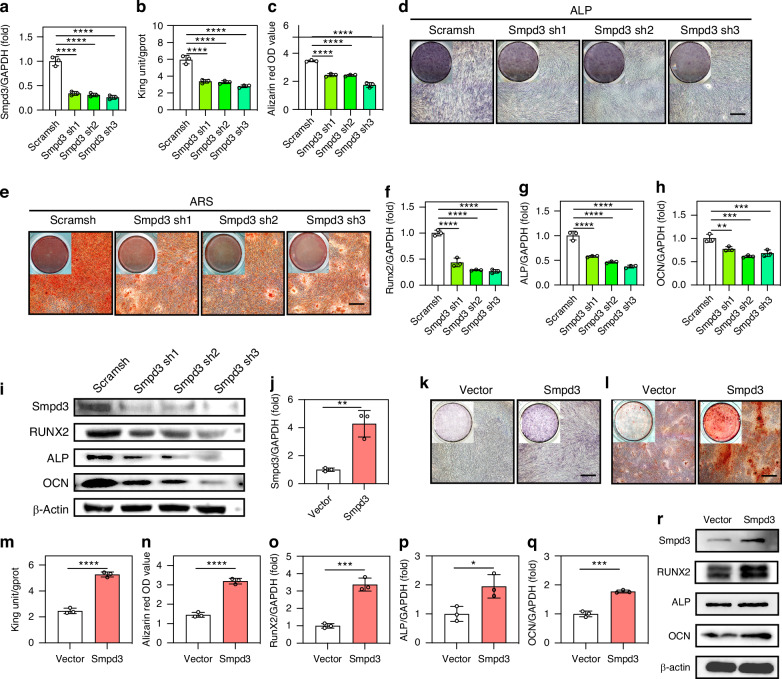
Smpd3 promotes osteogenic differentiation of jawbone-derived BMSCs. **a** Transfection efficiency of the Smpd3 knockdown virus. **b** After Smpd3 knockdown, the ALP activity decreased after 3 days of osteogenic induction (*n* = 5). **c** After Smpd3 knockdown, the calcium ion level significantly decreased after 14 days of osteogenic induction (*n* = 5). **d** After Smpd3 knockdown, the ALP staining decreased after 7 days of osteogenic induction (*n* = 5). **e** After Smpd3 knockdown, the ARS staining decreased after 14 days of osteogenic induction, with statistically significant differences (*n* = 5). **f**–**h** After Smpd3 knockdown, the mRNA expression levels of RUNX2, ALP, and OCN decreased after 7 days of osteogenic induction. **i** Western blot results showed that after Smpd3 knockdown, the protein expression levels of RUNX2, ALP, and OCN significantly decreased after 10 days of osteogenic induction (*n* = 5). **j** Transfection efficiency of Smpd3 overexpression virus. **k**, **l** After overexpression of Smpd3, ALP staining increased after 7 days of osteogenic induction, and ARS staining increased after 14 days of osteogenic induction, with statistically significant differences (*n* = 5). **m** After overexpression of Smpd3, the ALP activity increased after 3 days of osteogenic induction (*n* = 5). **n** After overexpression of Smpd3, the calcium ion level significantly increased after 14 days of osteogenic induction (*n* = 5). **o**–**q** After overexpression of Smpd3, the mRNA expression levels of RUNX2, ALP, and OCN increased after 7 days of osteogenic induction. **r** Western results showed that after overexpression of Smpd3, the protein expression levels of RUNX2, ALP, and OCN significantly increased after 10 days of osteogenic induction (*n* = 5) (scale bar = 50 μm, *P < 0.05, **P < 0.01, ***P < 0.001, ****P < 0.000 1 using one-way ANOVA and Student-Newman-Keuls test)

To further clarify the differential expression of Smpd3 in jawbone-derived BMSCs from T2DM patients, we included 15 T2DM patients and matched them with non-T2DM patients. Their jawbone bone debris was collected, and primary cultured jawbone-derived BMSCs were found to have low expression of Smpd3 at the protein (Fig. S1A) and mRNA (Fig. S1B) levels. Then, we transfected human jawbone-derived BMSCs with Smpd3 knockdown lentivirus and validated the knockdown efficiency (Fig. 1a). Using osteogenic induction medium to induce osteogenic differentiation of BMSCs, we found that Smpd3 knockdown reduced ALP activity (Fig. 1b) and calcium ion levels (Fig. 1c) in BMSCs and staining (Fig. 1d, e). The real-time RT‒PCR results showed that Smpd3 knockdown reduced the expression of the osteogenic markers Runx2 (Fig. 1f), ALP (Fig. 1g), and OCN (Fig. 1h) compared to that in the carrier group. In addition, immunoblotting revealed that Smpd3 knockdown decreased the protein levels of the osteogenic differentiation markers Runx2, ALP, and OCN (Fig. 1i). We validated the efficiency of the Smpd3-overexpressing lentivirus (Fig. 1j). With osteogenic induction medium, BMSCs overexpressing Smpd3 were induced to differentiate into osteoblasts. Overexpression of Smpd3 increased the expression of the osteogenic markers Runx2, ALP, and OCN in BMSCs, as determined by staining with ALP (Fig. 1k), Alizarin Red (Fig. 1l) and quantification of the ALP activity (Fig. 1m) and calcium ion levels (Fig. 1n). The real-time RT‒PCR results showed that Smpd3 overexpression increased the expression of the osteogenic markers Runx2 (Fig. 1o), ALP (Fig. 1p), and OCN (Fig. 1q) compared with those of the vector group. In addition, immunoblotting revealed overexpression of Smpd3 and increased protein levels of the osteogenic differentiation markers Runx2, ALP, and OCN (Fig. 1r). To further verify whether exogenous Smpd3 has a similar role, we added 25 µm and 50 µm of Smpd3 recombinant proteins to BMSC osteogenic induction medium. Runx2, ALP, and OCN were upregulated at the protein level with increasing Smpd3 protein concentration (Fig. S1C), and Alizarin Red mineralization was enhanced (Fig. S1D).

### Smpd3 mediates osteogenic differentiation of BMSCs through autophagy

**Fig. 2:**
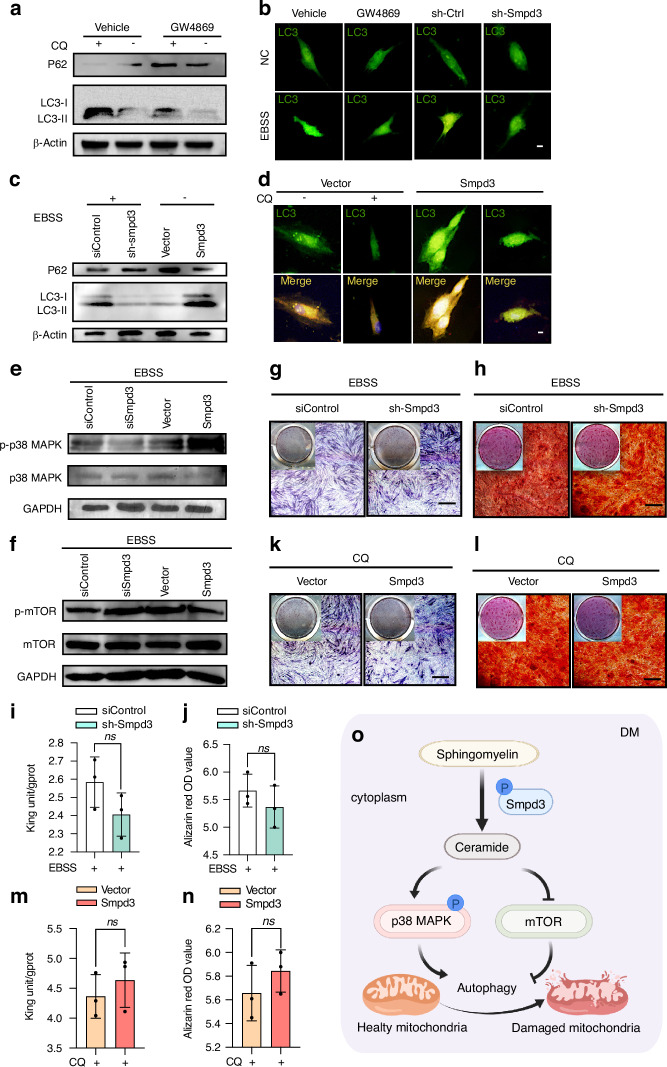
Smpd3 mediates osteogenic differentiation of BMSCs through autophagy. **a** After pretreatment with 50 μmol/L GW4869 (Smpd3 inhibitor) for 1 h to inhibit Smpd3 in the presence or absence of CQ. **b** Treatment with 5 μmol/L GW4869 and Smpd3 knockdown lentivirus inhibited Smpd3 expression, and LC3 puncta were assessed using immunofluorescence analysis. Smpd3 inhibition reduced the formation of LC3 puncta (green dots in the figure). **c** Overexpression of Smpd3 increased autophagic flux. Smpd3 knockdown downregulated LC3 and upregulated P62, while overexpression of Smpd3 upregulated LC3 and downregulated P62. **d** Direct overexpression of Smpd3 can induce 50 μmol/L MCQ to inhibit LC3 autophagic flux and increase LC3 puncta. **e** Smpd3 knockdown inhibited the expression of p-p38 MAPK induced by 1xEBSS (autophagy inducer), while overexpression of Smpd3 activated the expression of p-p38 MAPK induced by 1xEBSS. **f** Smpd3 knockdown activated 1xEBSS-induced p-p38 MAPK expression, while overexpression of Smpd3 inhibited 1xEBSS-induced p-p38 MAPK expression. **g**, **h** After activation of autophagy with 1xEBSS, shSmpd3 increased bone formation at 7 days and ARS staining at 14 days. **i**, **j** There was no significant difference in ALP and calcium ion levels between the osteogenic induction group and the siControl group at 3 days and 14 days. **k**–**n** After inhibition of autophagy by 50 μmol/L CQ, Smpd3 overexpression increased in ALP staining at 7 days of bone induction and ARS staining at 14 days of bone induction, with significant difference in ALP quantification and calcium ion quantification at 3 days of bone induction compared to those of the vector group. **o** Smpd3-mediated autophagic induction is achieved by activating p38 MAPK, inhibiting mTOR and promoting osteogenic differentiation of BMSCs by inducing autophagy (scale bar = 50 μm, *P < 0.05, **P < 0.01, ***P < 0.001, ****P < 0.000 1 using one-way ANOVA and Student-Newman-Keuls test)

To further investigate whether Smpd3 promotes osteogenic differentiation of jawbone-derived BMSCs through autophagy, we compared the levels of the autophagosome marker LC3-II in the presence or absence of the lysosomal inhibitor chloroquine (CQ) to estimate the degree of autophagic LC3 degradation. First, the inhibitory effect of the Smpd3 inhibitor GW4869 on Smpd3 at the protein and RNA levels was determined (Fig. S2A–C). Then, GW4869 was used to inhibit the expression of LC3 and increase the expression of P62 (Fig. 2a). LC3 immunofluorescence analysis also showed that GW4869 had a similar effect to shSmpd3 (Fig. 2b). With EBSS (autophagy activator), Smpd3 knockdown inhibited the expression of LC3 and increased the expression of P62, while without EBSS, Smpd3 overexpression significantly increased the expression of LC3 and inhibited the expression of P62 (Fig. 2c). Immunofluorescence revealed that LC3 was inhibited by the lysosomal inhibitor CQ, while overexpression of Smpd3 increased the formation of LC3 puncta (Fig. 2d). Smpd3 knockdown reduced the expression of p-p38 MAPK induced by EBSS, and overexpression of Smpd3 enhanced the expression of p-p38 MAPK induced by EBSS (Fig. 2e), while Smpd3 knockdown enhanced the expression of p-mTOR induced by EBSS. Overexpression of Smpd3 inhibited the expression of p-mTOR induced by EBSS (Fig. 2f), indicating that Smpd3 activated p38 MAPK and inhibited mTOR signal transduction. Functionally, after the addition of the autophagy inducer EBSS, Smpd3 knockdown weakened the inhibitory effect of BMSCs on osteogenic differentiation, including the differences in ALP staining (Fig. 2g), ARS staining (Fig. 2h), ALP activity (Fig. 2i), and calcium ion quantification (Fig. 2j). Similarly, under CQ, overexpression of Smpd3 weakened the enhancing effect of BMSCs on osteogenic differentiation, including the differences in ALP staining (Fig. 2k), ARS staining (Fig. 2l), ALP activity (Fig. 2m), and calcium ion quantification (Fig. 2n). Smpd3 induces autophagy in BMSCs through activation of p38 MAPK and inhibition of mTOR (Fig. 2o).

### Exos-Smpd3@Ns have good mechanical properties

**Fig. 3:**
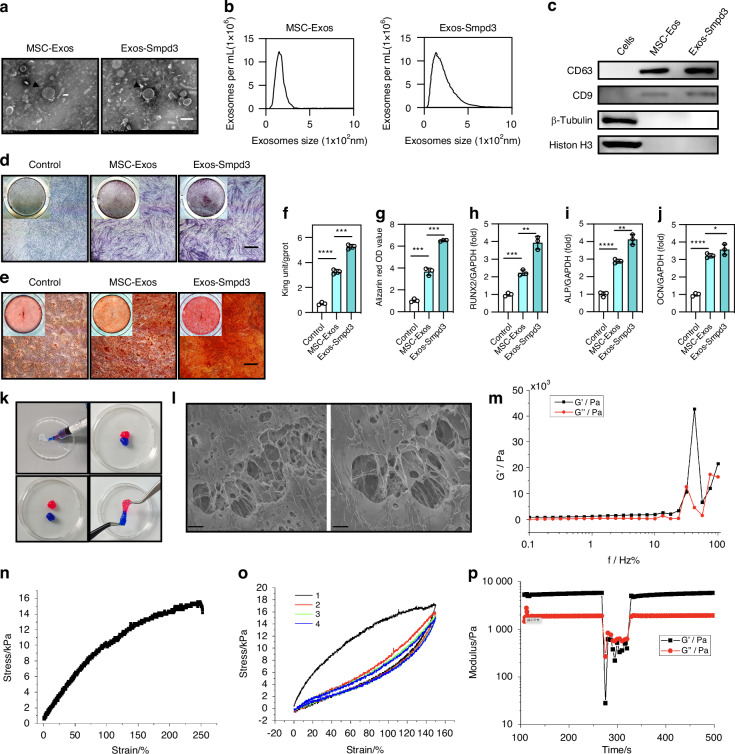
Exos-Smpd3@Ns have good mechanical properties. **a** SEM analysis of normal BMSC exosomes and BMSC exosomes overexpressing Smpd3, with black arrows indicating the characteristic disc-shaped vesicular structure of exosomes, at a scale of 100 nm. **b** Detection of extracellular vesicle particle size. **c** Identification of extracellular vesicle-specific protein markers. **d**, **e** Compared with 100 µg/µL normal BMSC exosomes, 1 µg/µL normal BMSC exosomes and PBS, the same dose of BMSC exosomes overexpressing Smpd3 enhanced ALP staining at 7 days of osteogenic induction and ARS staining at 14 days of osteogenic induction, with a scale of 50 μm. **f**, **g** Compared with 100 µg/µL normal BMSC exosomes, 1 µg/µL normal BMSC exosomes and PBS, the same dose of BMSC exosomes overexpressing Smpd3 enhanced ALP and calcium ion levels of BMSCs after 3 days of osteogenic induction, and the difference was statistically significant (*n* = 5). **h**–**j** Overexpression of Smpd3 in BMSCs enhanced osteogenic induction by extracellular vesicles for 7 days, and the change in the expression of osteogenic marker genes (Runx2, ALP, OCN) was statistically significant (*n* = 5). **k** The composite hydrogel is injectable and moldable. The hydrogel samples were dyed with red and blue dyes. **l** The SEM image shows the microstructure of the hydrogel, scale = 10 μmol/L and 5 μmol/L. **m** The oscillation frequency scan test showed the frequency dependence of the Exos-Smpd3@Ns on the storage and loss modulus. **n** The stress‒strain curves of different hydrogels under tensile load at a fixed strain rate of 0.411/s. **o** Hysteresis curve of the composite hydrogel under tensile loading and unloading. **p** The representative rheological measurements show the self-healing behavior of hydrogels during destructive shear and recovery. (*P < 0.05, **P < 0.01, ***P < 0.001, ****P < 0.000 1 using one-way ANOVA and Student-Newman-Keuls test.)

We first extracted exosomes from normal BMSCs and BMSCs overexpressing Smpd3 and characterized the exosomes, including their yield, morphology, typical surface protein expression, size distribution, and diameter. The exosomes were observed under transmission electron microscopy (TEM) to examine their size, shape, and morphology (Fig. 3a). The average particle sizes of exosomes from normal BMSCs and BMSCs overexpressing Smpd3 were 154 and 135 nm, respectively (Fig. 3b). Western blot analysis showed that compared to parental cells, all exosomes were rich in typical exosome CC surface proteins (CD63 and CD9) and lacked histone H3 and β-tubulin expression (Fig. 3c). In summary, these findings indicate that exosomes were successfully extracted from BMSCs. Subsequently, normal BMSC-derived exosomes and Smpd3-overexpressing BMSC-derived exosomes were added to the osteogenic induction medium, with PBS as the control. We found that Smpd3-overexpressing BMSC-derived exosomes could enhance ALP (Fig. 3d) and ARS staining (Fig. 3e) of BMSCs, improve ALP activity (Fig. 3h) and calcium ion levels (Fig. 3g), and increase the expression of the osteogenic markers RUNX2 (Fig. 3h), ALP (Fig. 3i), and OCN (Fig. 3j) at the mRNA level. In summary, we confirmed that overexpressing Smpd3 in BMSC-derived exosomes can significantly promote the osteogenic differentiation of BMSCs.

Next, we prepared an intelligent blood glucose fluctuation-responsive hydrogel delivery system, Exos-Smpd3@Ns. The injectability and plasticity of the composite hydrogel were shown by hydrogel staining (Fig. 3k). During scanning electron microscopy (SEM) analysis, the composite hydrogel showed a porous network with interconnected gelatin nanoparticles located in the polymer matrix (Fig. 3l). The oscillation frequency scan test showed that Exos-Smpd3@Ns had a frequency dependence on the storage and loss modulus (Fig. 3m). The tensile compression experiment showed that Exos-Smpd3@Ns have a certain degree of scalability and elasticity (Fig. 3n). The hysteresis curve indicates that Exos-Smpd3@Ns possess a strong denaturation ability (Fig. 3o). Representative rheological measurements showed that Exos-Smpd3@Ns exhibit self-repair behavior during destructive shearing and recovery processes and demonstrate substantial elasticity and toughness while exhibiting high deformability during shearing (Fig. 3p).

### Exos-Smpd3@Ns dynamically release and promote osteogenic differentiation of BMSCs in a blood glucose-fluctuating environment

**Fig. 4:**
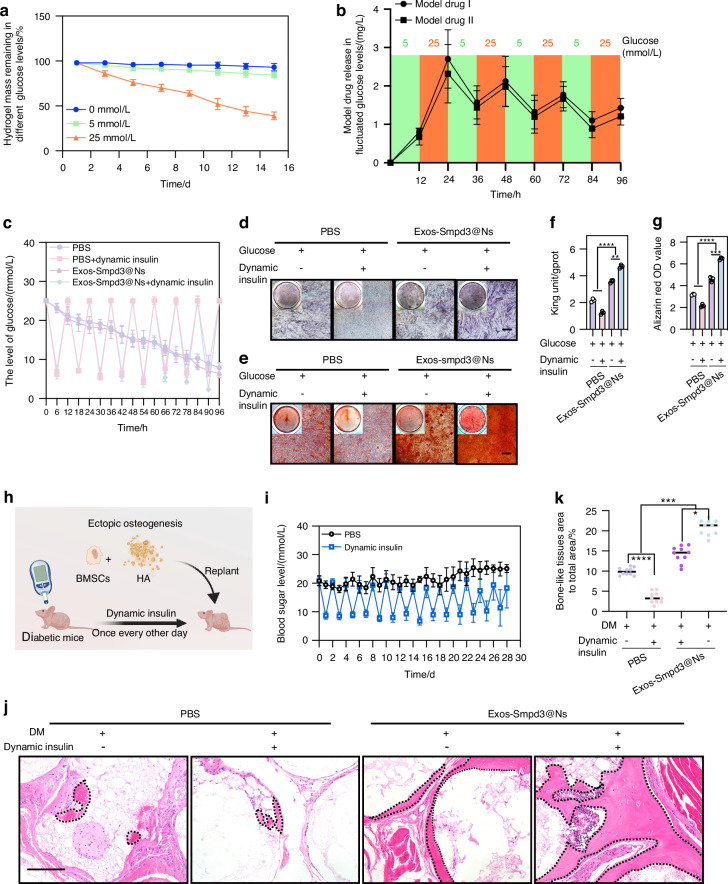
Exos-Smpd3@Ns show dynamic release and promote osteogenic differentiation of BMSCs in a blood glucose-fluctuating environment. **a** The degradation rate of the composite hydrogel at 5 mM and 25 mM glucose concentration. **b** Degradation curve of composite hydrogel in dynamic glucose interactive environment. **c** The sugar concentration of the culture medium was adjusted during the process of osteogenic induction through insulin, and the dynamic changes in sugar concentration were recorded. **d**, **e** Fluctuations in blood sugar significantly inhibit ALP staining at 7 days of osteogenic induction and ARS staining at 14 days of osteogenic induction in BMSCs, while Exos-Smpd3@Ns will have a salvage effect, scale = 50 μm. **f**, **g** Fluctuations in blood sugar significantly inhibit the quantification of ALP at 3 days of osteogenic induction and the quantification of calcium ions at 14 days of osteogenic induction Exos-Smpd3@Ns plays a salvage role. **h** Further validation using nude mouse replantation experiments with Exos-Smpd3@Ns. The effect of ectopic osteogenesis of BMSCs during blood glucose fluctuations. **i** The blood glucose levels in STZ-treated nude mice were regulated by fluctuating insulin injection, and changes in blood glucose levels were monitored within 30 days. **j** HE staining showed that the area of osteoid tissue (marked by a black dashed line) was smaller in the blood glucose fluctuation group and larger in the Exos-Smpd3@Ns group, scale = 25 μm. **k** During blood sugar fluctuations, the area of the bone-like tissue in the Exos-Smpd3@Ns group was the highest, and the difference was statistically significant. (Osteogenesis was shown to be induced by ALP quantification after 3 days, ALP staining after 7 days, and ARS staining and calcium ion quantification after 14 days, *P < 0.05, **P < 0.01, **P < 0.001, ****P < 0.000 1 using one-way analysis of variance and Student-Newman-Keuls test)

We first clarified the effect of glucose concentration on the osteogenic differentiation of jawbone-derived BMSCs. Under glucose concentrations of 0, 5, 25, 50, and 100 mmol/L, BMSCs were induced to undergo osteogenic differentiation. We found that a high glucose solution of 25 mmol/L had inhibitory effects on ARS and ALP staining, ALP activity, calcium ion levels, and mRNA and protein levels of the osteogenic markers RUNX2, ALP, and OCN in BMSCs, and the differences were statistically significant (Fig. S3A–E). Moreover, PI staining and flow cytometry showed that a 25 mmol/L high glucose solution significantly induced BMSC apoptosis (Fig. S4A, B). Therefore, a high glucose solution of 25 mmol/L was selected as the upper limit of blood glucose fluctuations, and the lower limit of blood glucose fluctuations was the normal glucose concentration in MSCM medium, which was 5 mmol/L. In a 25 mmol/L high sugar solution for 15 days, Exos-Smpd3@Ns degraded glucose faster, while the degradation rate was slower in a normal glucose solution of 5 mmol/L (Fig. 4a). In addition, we observed the release kinetics of simulated drugs I and II in a blood glucose fluctuation environment of 5 and 25 mmol/L. The simulated drugs were released in large quantities at a 25 mmol/L glucose concentration and in small quantities at a 5 mmol/L glucose concentration (Fig. 4b). To further verify Exos-Smpd3@Ns, we first established an in vitro blood glucose fluctuation environment by adding insulin to high glucose culture medium every other day to regulate the osteogenic differentiation of BMSCs in vitro. Moreover, we induced osteogenic differentiation of BMSCs. Figure 4c shows the glucose spectrum of the in vitro blood glucose fluctuation model. We found that compared to those of the conventional high glucose group, blood glucose fluctuations significantly inhibited the ARS and ALP staining of BMSCs, while Exos-Smpd3@Ns significantly enhanced the ALP (Fig. 4d) and ARS staining (Fig. 4e) of BMSCs, and similar results were found in ALP activity (Fig. 4f) and calcium ion levels (Fig. 4g), indicating that Exos-Smpd3@Ns significantly promote the osteogenic differentiation of BMSCs under blood glucose fluctuations. To further verify the effect of Exos-Smpd3@Ns on the heterotopic osteogenesis of BMSCs in a blood glucose fluctuation environment, we used streptozotocin (STZ) to establish a nude mouse model of diabetes and injected insulin every other day to establish a blood glucose fluctuation-type heterotopic osteogenesis model. Exos-Smpd3@Ns were implanted subcutaneously into nude mice after mixing, as shown in Fig. 4h. Figure 4i shows the blood glucose records of the STZ-treated nude mice within 1 month after insulin injection. We found that blood glucose fluctuations had a significant inhibitory effect on the ectopic osteogenesis of BMSCs, with a smaller area of osteoid tissue. Exos-Smpd3@Ns significantly increased the area of ectopic osteogenic osteoid-like tissue in BMSCs, and the osteoid-like tissue is marked with black dashed lines (Fig. 4j). The statistical results indicated that Exos-Smpd3@Ns significantly increased the ectopic osteogenic area of BMSCs under blood glucose fluctuations, and the difference was statistically significant (Fig. 4k).

### Exos-Smpd3@Ns promote M2 polarization of macrophages and inhibit inflammation in a blood glucose fluctuation environment

**Fig. 5:**
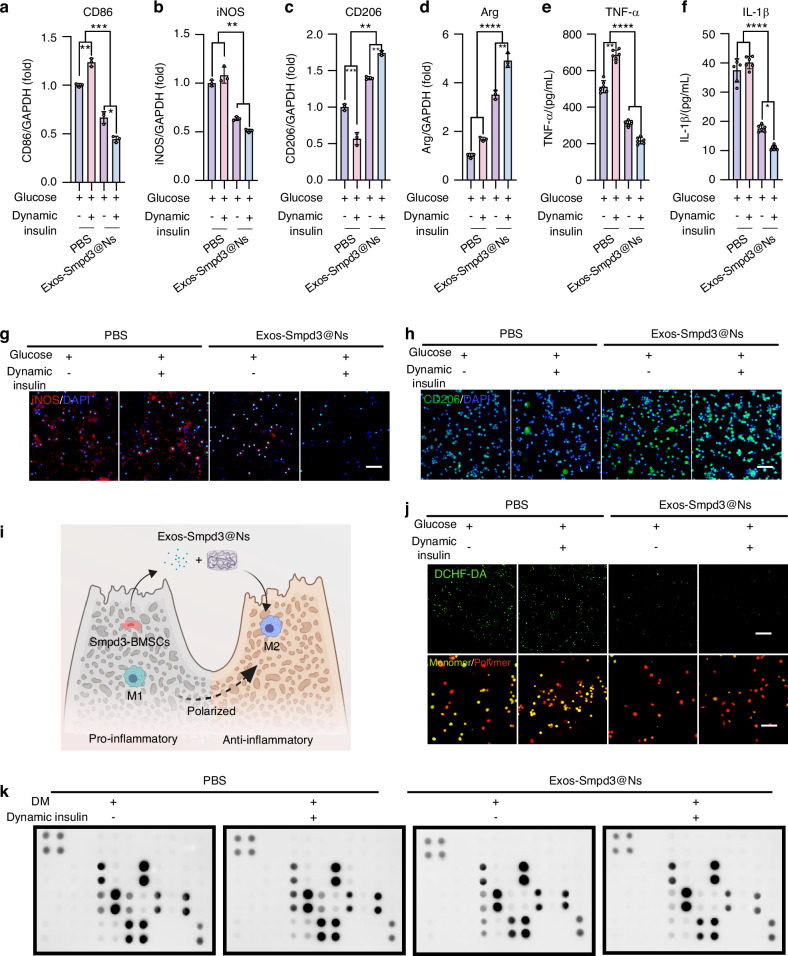
The effects of Exos-Smpd3@Ns on polarization and inflammation-related gene expression of macrophages in vitro. **a**–**d** Exo-Smpd3@N-mediated expression of iNOS, CD86, CD206, or Arg genes in macrophages under DM conditions. **e**, **f** ELISA determination of inflammatory cytokine (IL-1β) and TNF-α secretion. **g**, **h** Fluorescence microscopy images of iNOS and CD206 immunofluorescence staining, scale bar = 50 μm. **i** Schematic diagram of the Exos-Smpd3@Ns-mediated polarization of macrophages in vitro. **j** DCFH-DA green fluorescence staining was used to detect oxidative stress-induced damage in macrophages, while fluorescence microscopy images of JC-1 showed activation of mitochondrial membrane potential in macrophages (marked in red), scale bar = 50 μm. **k** Inflammatory factor protein array with Exos-Smpd3@Ns. The impact of treatment on the immune microenvironment is detailed in the supplementary images, which indicate the distribution of inflammatory factors, including IL-16, IP-10 (CXCL10), MCP-1, MCP-2, etc. These levels decreased after Exos-Smpd3@Ns treatment. (*P < 0.05, **P < 0.01, **P < 0.001, ****P < 0.000 1 using one-way ANOVA and Student-Newman-Keuls test)

To further explore the mechanism by which Exos-Smpd3@Ns promote the osteogenic differentiation of BMSCs in a blood glucose fluctuation environment, we simulated the relationship between human bone marrow-derived macrophages and blood glucose fluctuation with Exos-Smpd3@N cocultivation. Through qRT‒PCR, the expression of the M1 biomarkers CD86 and iNOS was shown to be significantly increased in the blood glucose fluctuation group, while Exos-Smpd3@Ns significantly reduced the expression of CD86 (Fig. 5a) and iNOS (Fig. 5b) while upregulating the expression of the M2 macrophage marker CD206 (Fig. 5c) and arginine (Arg) genes (Fig. 5d), indicating that Exos-Smpd3@Ns can inhibit the proinflammatory polarization caused by blood glucose fluctuations. In addition, enzyme-linked immunosorbent assay (ELISA) analysis showed that Exos-Smpd3@Ns significantly reduced the inflammatory cytokines TNF-α (Fig. 5e) and IL-1β (Fig. 5f) in macrophages. These anti-inflammatory functions may contribute to the effects of Exos-Smpd3@Ns. When faced with fluctuations in blood sugar and the production of a large amount of MMP, gelatin nanospheres decompose under protease treatment, thereby achieving efficient and safe delivery of Ag+. The immunofluorescence staining of macrophages was also affected by Exos-Smpd3@Ns. The promotion of anti-inflammatory polarization was verified, and in a blood glucose fluctuation environment, iNOS staining was increased, while CD206 staining was decreased. Exos-Smpd3@Ns significantly reduced the staining of M1-type iNOS (Fig. 5g) macrophages following blood glucose fluctuations and enhanced the staining of M2-type CD206 (Fig. 5h). As shown in Fig. 5i, Exos-Smpd3@Ns could reshape the bone immune microenvironment of alveolar bone macrophages in the context of blood glucose fluctuations. We polarized M1 macrophages exposed to blood glucose fluctuations into M2 macrophages with anti-inflammatory functions. In addition, we used DCFH-DA immunofluorescence staining to detect the production of ROS in macrophages. Under high ROS caused by blood glucose fluctuations, Exos-Smpd3@Ns also produced a significant clearing effect, and the differences in mitochondrial membrane potential between different groups were measured by JC-1 fluorescence staining as an indicator of mitochondrial function. Blood glucose fluctuations can lower the mitochondrial membrane potential, and Exos-Smpd3@Ns maintain the mitochondrial membrane potential (Fig. 5j). To further assess the effects of Exos-Smpd3@Ns, we performed dot blot analysis on human macrophages to evaluate their anti-inflammatory effects. The cytokines corresponding to the cytokine antibody chip are shown in Fig. S5A, and high expression of proinflammatory factors such as IL-6 and IP-10 was observed in the blood glucose fluctuation group with Exos-Smpd3@Ns, which reduced the expression of proinflammatory cytokines such as IL-6, IP-10, MCP-2, and MIG (Fig. 5k). Then, the bioinformatics analysis, including Gene Ontology (GO) analysis and Kyoto Encyclopedia of Genes and Genomes (KEGG) enrichment pathway analysis, showed that blood glucose fluctuations significantly increased the expression of signaling pathways such as Toll-like receptor and TNF cytokines. Exos-Smpd3@Ns relieved inflammation by inducing relevant pathways (Fig. S5B, C).

### Exos-Smpd3@Ns promote jawbone repair in DM model dogs

**Fig. 6:**
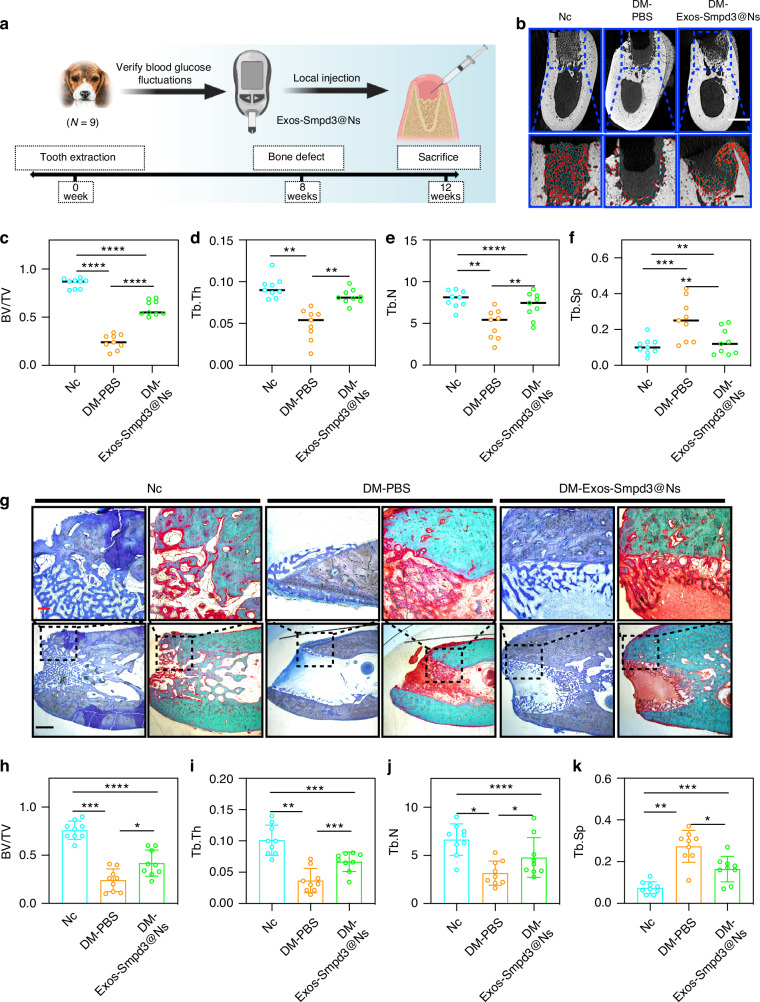
Exos-Smpd3@Ns promote jawbone repair in DM model dogs. **a** Schematic diagram of modeling time points for the blood glucose fluctuation dog model. **b** Micro-CT evaluation of Exos-Smpd3@Ns. The effect of treating blood glucose fluctuations in canine bone defects, with red dots indicating new bone formation. Scale bar = 5 mm and 1 mm. **c**–**f**. Micro-CT analysis of the indicators of BV/TV, Tb. Th, Tb. N, and Tb. Sp. **g** Histological staining, toluidine blue staining, and Goldner’s trichrome staining showed that blood glucose fluctuations in dogs resulted in almost no significant osteogenesis, and there was infiltration of red fibrous tissue. After Exos-Smpd3@Ns treatment, there was significant new bone formation (dark blue). Scale bar = 250 μm and 1 mm. **h**–**k**. The detection indicators of BV/TV, Tb. Th, Tb. N, and Tb. Sp in histological staining. (*P < 0.05, **P < 0.01, **P < 0.001, ****P < 0.000 1 using one-way ANOVA and Student-Newman-Keuls test)

We first established a diabetic beagle model, used irregular insulin treatment to control the blood sugar of beagles, and detected the blood sugar fluctuation of beagles within 1 month. We found that the blood sugar fluctuation range of diabetic beagles 2 h after a meal was 11.1–30.33 mm (Fig. S6A), which indicated that a large animal model with blood sugar fluctuation was successfully established. Then, a model of alveolar bone defect was established, and the surgical process is shown in Fig. S7A–F. Two months after the extraction of the teeth of a beagle, an animal model of jawbone defect was established, and Exos-Smpd3@Ns were injected locally at the bone defect site. A schematic diagram of the material collection after 4 weeks is shown (Fig. 6a). By evaluating the effect of bone defect repair through micro-CT, we found that blood glucose fluctuations have a significant inhibitory effect on new bone formation. Exos-Smpd3@Ns increased the area of new bone formation, with the red dots indicating new bone (Fig. 6b). Statistical analysis showed that blood glucose fluctuations significantly reduced bone volume/total volume ratio (BV/TV) (Fig. 6c), trabecular thickness (Tb. Th) (Fig. 6d), and number of trabeculae (Tb. N) (Fig. 6e) and increased trabecular spacing (Tb. Sp) (Fig. 6f), and Exos-Smpd3@Ns increased BV/TV, Tb. Th, and Tb. N and reduced Tb. Sp, although the levels did not reach those of the control group. Toluidine blue staining and Goldner’s trichrome staining showed that the negative control group could form a complete woven bone-like structure at 1 month, while the blood glucose fluctuation group showed almost no significant new bone formation and a large amount of fibrous tissue infiltration. The Exos-Smpd3@Ns group had an increased area of new bone formation (Fig. 6g). In addition, the statistical results were similar to those of micro-CT, where blood glucose fluctuations significantly reduced BV/TV (Fig. 6h), Tb. Th (Fig. 6I), and Tb. N (Fig. 6j) and increased Tb. Sp (Fig. 6k), and Exos-Smpd3@Ns increased BV/TV, Tb. Th, Tb. N and reduced Tb. Sp, although the levels did not fully reach the control group levels.

### Schematic diagram of the repair of diabetic bone defects by Exos-Smpd3@Ns

**Fig. 7:**
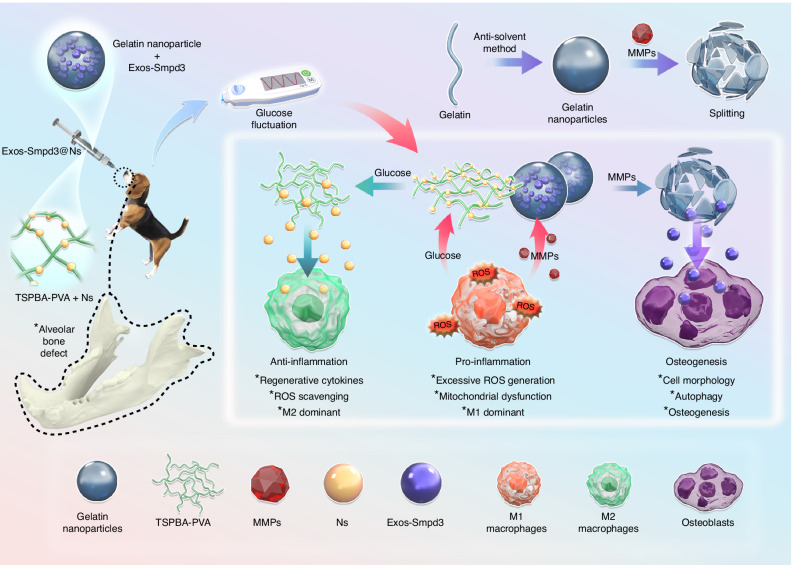
Based on the degradability of gelatin nanospheres on MMPs, Exo Smpd3-overexpressing Smpd3 exosomes were encapsulated, and the responsiveness of TSPBA-PVA to glucose was utilized to load nanosilver ions with safe and efficient anti-inflammatory effects, which were tested after establishment of a blood glucose fluctuation-type jawbone defect in beagle dogs. In the jawbone defect, TSPBA-PVA released silver ions under high sugar, which had an anti-inflammatory effect, promoted macrophage polarization from M1 to M2, and displayed anti-ROS effects. Gelatin nanospheres release Exo Smpd3 with MMPs, promoting bone regeneration

In summary, we extracted BMSC exosomes expressing Smpd3, encapsulated them in gelatin nanospheres, and then loaded silver ions into TSPBA-PVA to obtain Exos-Smpd3@Ns. In vitro and in vivo, Exos-Smpd3@Ns exhibited good anti-inflammatory, macrophage polarization, and osteogenic effects (Fig. 7).

## Discussion

In recent years, researchers have gradually discovered that ceramides play a crucial role in the pathophysiological processes of metabolic syndromes such as T2DM. The production of ceramides is inseparable from the catalytic effect of Smpd3, and the dysregulation of these molecules seems to be the basis of many inflammation-related pathologies, including the inhibition of Smpd3-produced ceramides, which accumulate in the endoplasmic reticulum (ER) and lead to ER stress. These changes in turn lead to the activation of c-Jun N-terminal kinase (JNK), thus inhibiting insulin receptor signaling, causing insulin resistance, metabolic dysfunction, cellular stress and inflammation, as well as mitochondrial degeneration and an increase in ROS secretion in cells, accompanied by defective glucose-stimulated insulin secretion.^25^ We also found that Smpd3 was downregulated in BMSCs of patients with diabetes with fluctuating blood glucose, which suggests that Smpd3 has potential research value in the pathological mechanism of T2DM. In this study, we first found that the expression of Smpd3 in jawbone-derived BMSCs from patients with diabetes was reduced, Smpd3 knockdown inhibited the osteogenic differentiation of BMSCs from patients with diabetes, and Smpd3 overexpression significantly promoted the osteogenic differentiation of BMSCs from patients with diabetes, indicating that Smpd3 plays a role in promoting the osteogenic function of jawbone-derived BMSCs from patients with blood glucose fluctuations. Smpd3 is an important regulator in bone and cartilage development and mineralization. A lack of SMPD3 can affect intramembrane and cartilage osteogenesis, and loss of SMPD3 activity can affect normal apoptosis of hypertrophic chondrocytes in developing bones.^20^ In addition, Smpd3 can promote the mRNA expression of ALP, type I collagen, and osteocalcin in periodontal ligament stem cells, and the Smpd3 inhibitor GW4869 significantly inhibits the expression of these osteogenic differentiation indicators, which is similar to our findings in jawbone-derived BMSCs.^21^ In addition, deletion of the Smpd3 gene leads to severe osteogenesis and dentinogenesis imperfection syndrome in mice, further indicating the potential role of Smpd3 in bone metabolism and development. In addition, our previous research demonstrated that the expression of Smpd3 is downregulated in bone tissue around implants in diabetic rats, indicating the need to further study the role of this enzyme in bone regeneration.^23,26^

Notably, our in vitro studies have shown that Smpd3 can mediate autophagy in BMSCs by activating p38 MAPK, inhibiting mTOR phosphorylation, and promoting osteogenic differentiation of BMSCs. Autophagy is an essential metabolic process in eukaryotic cells that maintains metabolic energy homeostasis and plays a crucial role in bone regeneration by controlling molecular degradation and organelle renewal. Mesenchymal cells exhibit autophagy during osteogenic differentiation, and the molecular mechanisms and targets involved are continuously being explored and updated, showing good application potential.^27^ Recent studies have shown that the catalytic product of Smpd3, ceramide, a membrane component that mediates membrane dynamics by changing fluidity or stiffness, plays a core role in sphingolipid metabolism and can act as a signaling molecule to induce autophagy. Under starvation conditions, Smpd3 quickly and stably activates enzymatic reactions in the Golgi apparatus, preventing cell damage.^28^ We found that Smpd3 knockdown lentivirus and the inhibitor GW4869 significantly inhibited LC3 puncta formation and promoted the degradation of the autophagic substrate p62. Overexpression of Smpd3 increased LC3 puncta formation and inhibited p62 degradation, indicating that Smpd3 activated autophagic flux. In addition, under autophagic inhibition with CQ, the osteogenic effect of Smpd3 was inhibited, while with the autophagy activator EBSS, the osteogenic effect of Smpd3 was promoted, indicating that autophagy is indeed related to the osteogenic differentiation of Smpd3. Autophagy regulators regulate autophagy, which is beneficial to bone regeneration, including mTOR inhibitors, AMPK activators and emerging phytochemicals. In terms of bone regeneration in diabetes, scholars found that PPAR β/δ activation can improve the osteogenesis and differentiation of BMSCs under high glucose conditions and promote bone regeneration in skull defects in diabetic rats, while PPAR β/δ inhibition can alleviate the osteogenic differentiation of BMSCs.^29^ As a potential target for stem cell therapy, autophagy can reduce the survival, proliferation, and function of immune cells involved in injury-induced inflammation and is beneficial for alleviating inflammation. Moreover, the impact of stem cells on autophagy is bidirectional. Stem cells can affect the autophagy of endogenous progenitor cells, promote their survival, proliferation, and differentiation, and support the recovery of functional tissues.^30^ In this study, we focused on the potential value of Smpd3 in autophagic activation and considered its use as a target to further enhance the endogenous autophagy levels of BMSCs, subsequently restoring damaged BMSC function and affecting bone regeneration in T2DM patients. This endogenous backbone prediction strategy has a high biosafety advantage. In addition, the mechanical role of ceramides in membrane dynamics may be a possible mechanism for Smpd3-mediated autophagic induction. Studies have shown that Smpd3 induces surface modification of the Golgi lipid membrane during the conversion of ceramides, mediating vesicle transport. However, the lack of Smpd3 may induce functional defects in the Golgi apparatus, leading to vesicle transport disorders.^31–33^ The Golgi apparatus provides membrane power to autophagosomes through the supply of vesicles. Further research is needed on the role of the Golgi apparatus in autophagy.

In addition, recent studies have shown that Smpd3-mediated autophagy can promote the secretion of exosomes.^34^ In this study, we used stem cell exosomes overexpressing Smpd3, which require no complex modifications compared to exosomes modified in vitro. As exosomes are close to natural components of the human body, these vesicles can effectively retain genetic information from the body. Compared with unmodified BMSC exosomes, Smpd3-overexpressing exosomes showed a significant promoting effect on the osteogenic differentiation of BMSCs, which also confirms the advantage of Smpd3-overexpressing extracellular vesicles over unmodified extracellular vesicles in osteogenesis.^35^ Given the nanovesicle properties and physiological functions of exosomes, compared to traditional “foreign” nanocarriers, stem cell exosomes can serve as a natural new type of nanocarrier, with the ability to target damaged lesions, tissue repair, good stability and biocompatibility, low immunogenicity, and the ability to mediate genetically modified cellular functions. Undoubtedly, the use of Smpd3-overexpressing BMSC exosomes for nanomodule content loading is an excellent choice.^36,37^ To construct a precise responsive delivery system, we utilized high glucose and high protease caused by blood glucose fluctuations to correspond to the switch of phenylboronic acid-based polyvinyl alcohol crosslinking agents and gelatin nanospheres, respectively. Through the reversibility of TSPBA-PVA degradation in glucose and the protease degradation of gelatin nanospheres, we generated Exos-Smpd3@Ns that can degrade and release effective factors at a high sugar level of 25 mM while retaining effective factors at a low sugar level of 5 mM, thereby achieving precise slow-release targets and improving repair effectiveness to the greatest extent possible.^24^ Additionally, we found that Exos-Smpd3@Ns have good mechanical properties, possess good viscoelasticity, and have potential value in addressing large or complex bone defects. In an in vitro blood glucose fluctuation environment, Exos-Smpd3@Ns promoted the osteogenic differentiation of BMSCs and effectively increased the ectopic osteogenic area of BMSCs in nude mice with blood glucose fluctuations. Notably, bone tissue engineering regeneration not only requires induction but also the guiding effect of the Exos-Smpd3@N scaffold, which has a sponge-like structure and can provide a certain space for the adhesion and proliferation of BMSCs, which is conducive to metabolism and signal transmission. Moreover, bone is an organ containing vascular tissue, and Exos-Smpd3@Ns have the potential to play a role in the regeneration of new blood vessels.^38^

Notably, at the beginning of this study, T2DM was considered a chronic inflammatory disease, and a long-term inflammatory state would accelerate the degradation of effective factors. Exos-Smpd3@Ns can exert long-term immune regulatory activity in bone regeneration. Exos-Smpd3@Ns can efficiently promote the upregulation of the M2 polarization markers CD206 and Arg in macrophages in an in vitro blood glucose fluctuation environment and can also inhibit the inflammatory factors TNF-*α* and IL-1, which inhibit bone regeneration and produce inhibitory effects. Exos-Smpd3@Ns show good antioxidant capacity, inhibit ROS generation and activate mitochondrial membrane potential in a blood glucose fluctuation environment. This activation ability may come from Exos-Smpd3@Ns. The carrier itself has a good effective concentration maintenance effect in a blood glucose fluctuation environment, the anti-inflammatory targeting ability of MSC Exos and the anti-inflammatory ability of nanosilver ions.^39^ As Exos-Smpd3, which transmit genetic information on Smpd3, may induce Smpd3 to inhibit high ROS, TNF-α, and IL-1 β induced by high sugar, the expression of Smpd3 can also activate the release of exosomes, and the positive feedback activation of Smpd3 exosomes may also promote the osteogenic differentiation potential of BMSCs with Smpd3 as the core.^40^

In addition to the role of exosomes themselves, the delivery system also includes components such as phenylboronic acid polyvinyl alcohol crosslinkers, nanosilver, and gelatin, which may be beneficial for bone regeneration. Nanosilver ions have excellent antibacterial effects, can bind with the cell wall/membrane of pathogenic bacteria, directly enter the bacterial body, quickly bind with the thiol (-SH) of oxygen metabolic enzymes, inactivate enzymes, block respiratory metabolism, and suffocate bacteria to death.^41^ Nanosilver has effective anti-inflammatory properties, targeting acute inflammation of macrophages in T2DM, and can inhibit immune inflammation caused by high sugar, promote polarization of M1 macrophages to M2 macrophages, and alleviate the inflammatory state caused by high sugar. These ions can kill broad-spectrum bacteria within a few minutes without any drug resistance, and low-concentration silver nanoparticles can quickly kill pathogenic bacteria.^42^ In addition, the carrier contains a phenylboronic acid polyvinyl alcohol crosslinker (TSPBA-PVA) that is responsive to blood glucose. Its sugar and reactive oxygen species-responsive ability can be used as a switch for therapeutic carriers. When the drug carrier enters the body, the reactive oxygen species increase when blood sugar increases, TSPBA-PVA is decomposed and effective factors are released, while when blood sugar decreases, reactive oxygen species decrease and reaggregation of TSPBA-PVA occurs to maintain the effective therapeutic concentration.^43^ In addition, gelatin has a response to matrix metalloproteinases. When blood sugar increases, matrix metalloproteinases increase in the bone repair defect area. At this time, matrix metalloproteinases can dissolve gelatin, thereby achieving responsive release of effective factors.^44^ In this study, Exos-Smpd3@Ns demonstrated excellent osteogenic effects, macrophage polarization from M1 to M2, ROS clearance, and the ability to reduce inflammatory factor expression, but for human studies of Exos-Smpd3@Ns, clinical trials must be conducted. In this study, using a beagle as a large animal model, we found that blood glucose fluctuations resulted in a poorer ability to heal jawbone defects in beagles in terms of imaging and histology. Exos-Smpd3@Ns effectively increased the healing of jawbones of dogs with blood sugar fluctuations and promoted the formation of new bones, but these effects did not reach the healing level of nondiabetic dogs. The possible reason is that although insulin is used to regulate the blood sugar of diabetic dogs, their blood sugar is still at a high level, which may not induce the sustained release of active ingredients of Exos-Smpd3@Ns. Beagle dogs are one of the most mature animal models in dental research. It is very important to use big animal models such as beagle dogs to simulate blood sugar fluctuations in patients with diabetes. Many dental studies use beagle dogs as animal models. For example, Maryam and others established beagle dog implant animal models and applied a doxycycline layer on TiZr implants, which were conducive to reducing or eliminating infection after implantation. Liu and others used beagle dog animal models. A new dental implant made of titanium copper alloy maintained a balance between aerobic and anaerobic bacterial communities, improving the implant’s ability to resist infections.^45,46^ The alveolar bone, periodontal tissue, and tooth size of beagle dogs are largely similar to those of humans. In this study, large beagle dogs were used as the research model with Exos-Smpd3@Ns. Preclinical trials provided effective evidence.^47^

This study focuses on the difficulty in repairing bone defects caused by blood glucose fluctuations in T2DM patients. First, Smpd3 was found to promote the osteogenic differentiation of BMSCs in T2DM patients through autophagy, and glucose-responsive TSPBA-PVA was used as a carrier switch. Based on the nonimmunogenic characteristics of extracellular vesicles, Smpd3-overexpressing MSC Exos achieve extracellular vesicle module loading, complete the coupling of nanosilver ions, and leverage Smpd3 to promote bone formation. Our research results provide a theoretical basis and candidate methods for the treatment of bone defects in T2DM patients by fully ensuring the construction of a nonimmunogenic biomimetic coordination system and improving the precise response of osteogenesis, immune regulation, and anti-inflammatory abilities in the environment of blood glucose fluctuations, thereby achieving precise regulation of the repair process of T2DM bone defects.

## Subject registration and ethical declaration

This study was approved by the Ethics Committee of Beijing Stomatological Hospital, Capital Medical University (Approval No.: CMUSH-IRB-KJ-YJ-2023-07), with informed consent from the patients. All participants were recruited by a surgeon from the dental implant center. All enrolled T2DM patients in this study were diagnosed with T2DM by endocrinologists, and all patients had HbA1c below 8%. Over the past 3 years, 15 patients who met blood glucose control standards and were preparing for implantation surgery were included in the study. Compared with those in the T2DM group, participants in the non-T2DM group were selected at a 1:1 ratio, and all basic information, including age, sex, general health status, DM type, and implant placement, was matched to avoid the influence of other factors. Patients with contraindications for implantation surgery, such as cardiovascular disease, kidney disease, or uncontrolled periodontitis, were excluded. The STROBE guidelines were followed to ensure the rigor of our study (Table S1).

### Cell culture

The primary culture method of human jawbone-derived BMSCs was based on a previous report from the research group.^48^ In short, implantation surgery was performed by a surgeon. Discarded bone fragments were collected during the preparation process of implantation surgery and stored in phosphate-buffered saline (Gibco, Grand Island, NY, USA) containing antibiotics (10 000 U/mL penicillin and 10 mg/mL streptomycin; Gibco). After centrifugation at 1 100 r/min, the bone fragments were resuspended in mesenchymal stem cell culture medium (MSCM; ScienCell, Carlsbad, CA, USA), inoculated into culture dishes, and cultured in a moist cell culture incubator containing 5% CO_2_ at 37 °C for 7 days without movement. A single-cell suspension was obtained using filters with a 70 μm pore size (Falcon, BD Labware, Wilmington, DE, USA) and then cultured in MSCM. The culture medium was changed every 3 days. Fourth-generation BSCMs were used in subsequent experiments. Primary cells obtained through low-speed drilling technology and cultured using the above methods exhibit BMSC characteristics.^49^ Human bone marrow-derived primary macrophages were purchased from Punosai Biotechnology Co., Ltd.

### Osteogenic induction and detection of BMSCs

P4 BMSCs were seeded at a rate of 5 × 10^5^ cells per well in a 6-well culture dish and cultured in osteogenic medium using the StemPro osteogenic differentiation kit (Invitrogen, Waltham, MA, USA) according to the manufacturer’s instructions for 14 days. Osteogenic differentiation was induced after the cells reached 70%–80% confluence. According to the manufacturer’s instructions, on the seventh day after osteogenic induction, the intracellular ALP activity in BMSCs was evaluated using an ALP activity assay kit (Nanjing Jiancheng Institute of Biotechnology, China) and standardized based on protein concentration. The optical density (OD) was recorded at 520 nm. After 14 days of osteogenic induction, the cells were fixed in 70% ethanol and stained with 2% ARS (Sigma-Aldrich, St. Louis, MO, USA) for 5 min. Then, 1 ml of isopropanol was added to each well to dissolve the red perylene quinone derivative in the calcium nodules, and the OD value was measured at 550 nm using a specific gravity meter (5111970DPC, Thermo Fisher Scientific, Waltham, USA). For the blood glucose fluctuation group, culture medium containing 25 mmol/L or 5 mmol/L glucose was exchanged every 12 h.

### Cell transfection

The Smpd3 knockdown lentivirus control and experimental lentivirus were fused with green fluorescent protein (GFP), namely, Scramsh and Smpd3 sh1-3, and the Smpd3 overexpression lentivirus control and experimental lentivirus, namely, vector and Smpd3, were purchased from Shanghai Jima Gene Biotechnology Co., Ltd. (Shanghai, China). These lentiviral constructs were stably transfected into BMSCs with a multiplicity of infection (MOI) of 50 using 5 μg/ml puromycin (Shanghai Jima Gene Biotechnology Co., Ltd.). After 18 h, the lentivirus medium was replaced with a fresh culture medium.

### Western blotting

Cell lysis was performed using radioimmunoprecipitation analysis (RIPA) buffer (Sigma-Aldrich). The protein was separated by 15% sodium dodecyl sulfate‒polyacrylamide gel electrophoresis (SDS‒PAGE) and transferred to a polyvinylidene fluoride membrane (Bio-Rad, Hercules, CA, USA) using a semidry transfer device (Bio-Rad). The membrane was sealed with 5% dehydrated milk in TBS-T (20 mmol/L Tris HCl, pH 7.6, 150 mmol/L NaCl, 0.05% Tween 20) for 1 h and then incubated with the primary antibody at 4 °C for 24 h. The primary antibodies used were anti-Smpd3 (A10197; 1:1 000, ABclonal), anti-Runx2 (A2851; 1:1 000, ABclonal), anti-ALP (ab307726; 1:1 000, Abcam), anti-OCN (ab133612; 1:1 000, Abcam), anti- β-actin (AC038; 1:10 000, ABclonal), anti-P62 (sc-28359; 1:1 000, Santa Cruz), anti-LC3A/LC3B (A5618; 1:1 000, ABclonal), anti-p-p38 MAPK (AP0526,1:1 000, ABclonal), anti-p38 MAPK (A14401; 1:1 000, ABclonal), anti-p-mTOR (AP0115; 1:1 000, ABclonal), anti-mTOR (A2445; 1:1 000, ABclonal), anti-GAPDH (AC002); 1:10 000, ABclonal, anti-CD63 (A19023; 1:1 000, ABclonal), anti-CD9 (A19027; 1:1 000, ABclonal), anti-β- tubulin (AC008; 1:1 000, ABclonal), and anti-histone H3 (A2348; 1:1 000, ABclonal). The secondary antibodies were HRP goat anti-rabbit IgG (AS014; 1:1 000, ABclonal) and HRP goat anti-mouse IgG (AS003; 1:1 000, ABclonal). The protein bands were visualized using Super Signal reagent (Bio-Rad).

### Reverse transcription and real-time quantitative PCR

TRIzol (Invitrogen) was used for total RNA isolation, and first-strand cDNA was synthesized using a reverse transcription system (TaKaRa, Kusatsu, Japan). The Power SYBR Green PCR Master Mix (Roche, Basel, Switzerland) and 7500 real-time PCR detection systems (Applied Biosystems, Foster City, CA, USA) were used to quantify all gene transcripts using quantitative real-time PCR. The following are the thermal cycling conditions: 95 °C for 10 min, followed by 40 cycles of 95 °C for 15 s and 60 °C for 1 min. The primers used are listed in Table S2. Data were analyzed using the 2−ΔΔCT method.

### Immunofluorescence

After fixation and infiltration, BMSCs were incubated overnight with primary antibodies under humid conditions at 4 °C. After PBS washes, the corresponding secondary antibody was added to the primary antibody for 1 h. The primary antibodies used in this study are listed in Table S1. After PBS washes, the cells were incubated with DAPI for 10 min to delineate the nucleus. Immunofluorescence images were captured using a fluorescence microscope (Leica, Germany). The cells were fixed in PBS with 4% formaldehyde for 20 min. The cells were washed with 0.3% Triton X-100 (TX-100) in PBS three times and incubated with 5% BSA and 0.3% Triton 100 at RT for 1 h. Then, the cells were incubated in PBST for 24 h using the following primary antibodies: anti-LC3A/LC3B (A5618, 1:100, ABclonal), anti-iNOS (A3774, 1:100, ABclonal), anti-CD206 (24595, 1:100, Cell Signaling), Alexa Fluor 488-conjugated goat anti-rabbit (AS053, 1:200, ABclonal), and Alexa Fluor 594-conjugated goat anti-rabbit (AS039, 1:200, ABclonal). After three washes, the cells were incubated with secondary antibodies at RT for 1 h. DAPI was used for nuclear staining, and after three washes, the fluorescence quenching agent was removed, and a cover glass was added. The cell images were obtained using confocal microscopy (LSM 710; Zeiss, Leica, Germany).

### Preparation of Exos-Smpd3@Ns

BMSCs were inoculated into T-75 culture bottles and passaged near confluence after trypsin digestion. The cells were incubated in MSCM containing 10% exosome-deficient FBS for another 3 days, and conditioned medium was collected, centrifuged at 3 000×*g* for 15 min, and passed through a 0.22 μm Sigma-Aldrich (USA) filtration unit to remove dead cells and debris. For concentration of the extracellular vesicles and remobilization of the proteins in the culture medium, the medium was centrifuged through an Ultra15 centrifugation filtration unit (100 kD, UFC910024) (Millipore, USA) according to the manufacturer’s protocol. Extracellular vesicles were directly used for downstream experiments. Next, we prepared a blood glucose fluctuation intelligent hydrogel delivery system, Exos-Smpd3@Ns. TSPBA-PVA, gelatin nanospheres, and silver nitrate, which were provided by Xi’an Qiyue Biotechnology Co., Ltd., were used to prepare phenylboronic acid-based polyvinyl alcohol crosslinkers using the method described in the literature.^24^ Specifically, a phenylboronic acid-based crosslinking agent, N1-(4-borobenzyl)-N3-(4-borophenyl)-N1, N1, N3, N3 tetramethylpropane-1,3-diamine (TSPBA), was synthesized by selecting PVA with a molecular weight of 145 kDa and reacting it with PVA. The phenylboronic acid (PBA) group of TSPBA can form a phenylboronic acid ester bond with the diol group of PVA. Moreover, a PBA/OH ratio of 0.5 was used, and silver nitrate was added. With a sodium borohydride reducing agent, silver nitrate was reduced to nanosilver ions, forming a complex nanosilver ion phenylboronic acid-based polyvinyl alcohol crosslinking agent (TSPBA-PVA), which contained a colloid volume fraction of 0.5 < φ < 0.64. Preparation of a high-concentration gelatin nanosphere suspension with a value of 0.64 was performed by direct mixing 1 µg/µl exosomes and TSPBA-PVA solution, yielding Exos-Smpd3@Ns.

### Analysis of Exos-Smpd3@Ns

SEM (Zeiss, Sigma 300, Germany) was used to observe the microstructure of Exos-Smpd3@Ns. Specifically, Exos-Smpd3@Ns were frozen and fractured at −80 °C and then freeze-dried to remove moisture. The cross section of the hydrogel sample was then coated with gold to improve conductivity. NanoBrook 90plus PALS (Brookhaven, USA) was used to determine the particle size of extracellular vesicles and prepare dumbbell-shaped samples for tensile testing. The quantified fracture energy (kJ/m^3^) obtained by calculating the area under the tensile stress‒strain curve was used to characterize the work needed to break the sample per unit volume. The elastic modulus was defined as the initial slope (10%) value of the stress‒strain curve. Rheology and adhesion testing are described in the Supplementary Information. The self-healing behavior of the hydrogel was evaluated by tensile testing. The hydrogel sample was cut into two separate blocks and then brought into direct contact. After 1 min of contact, we evaluated the sample using a tensile test. The adhesive strength (*n* = 5) characterized by the lap shear stress of the hydrogel was recorded.

### Detection of inflammatory factors

We used a TNF-α/IL-1β Quantikine ELISA kit (R&D, USA) to evaluate TNF-α and IL-1β. Analysis of 41 inflammatory factors in macrophages was performed using an AAH-INF-3 antibody membrane chip (Raybiotech, USA). All test kits were used according to the manufacturer’s instructions.

### Animals and surgical procedures

The nude mouse experiment was approved by the Animal Care and Use Committee of Beijing Stomatological Hospital (KQYY-202211-007). The animal experiments on beagle dogs were approved by the Animal Care and Use Committee of Beijing Stomatological Hospital (KQYY-202211-007). This study complies with the guidelines of Animal Research: In vivo Experimental Reports of Preclinical Animal Research (Appendix), and approval was obtained. All animal experiments were conducted in accordance with institutional guidelines.

Forty-five-week-old male BALB/C homozygous nude mice (nu/nu) (SPF Biotechnology Co., Ltd., Beijing, China) were randomly divided into four groups with 10 mice in each group. All mice were treated with STZ (Sigma, USA) dissolved in citric acid trisodium citrate buffer solution (0.1 mol/L pH 4.5) to prepare a 1% injection. The sample was filtered by a 0.22 µm filter and intraperitoneally injected at a dose of 150 mg/kg, and a diabetic diet was given. Blood glucose levels were measured every 3 days after injection through the tail vein. When the average blood glucose level was greater than 16.7 mmol/L, the modeling standard was reached. Then, insulin was used for the blood glucose fluctuation group. Insulin (0.5 U/mL) was injected the next day, with a subcutaneous injection rate of 0.054 mL per 10 g. Blood glucose levels were measured for 2 h. When blood glucose significantly decreased, blood glucose fluctuations were considered to have occurred. In 1 month, the blood glucose dynamics in the blood glucose fluctuation group of nude mice were determined and are shown in Fig. 5g. The steps for transplanting nude mice were the same as described previously.^49^ In short, a single-cell suspension of P4 BMSCs was obtained after trypsin digestion, and the cell density was adjusted to 4 × 10^6^ cells per mL. The cells were incubated with 40 mg of hydroxyapatite bone powder at 37 °C for 1 h and centrifuged at 150×*g* for 5 min at 37 °C. The processed cells and control cells were implanted into the subcutaneous space on the back of 5-week-old female BALB/C homozygous nude mice (nu/nu) (SPF Biotechnology Co., Ltd., Beijing, China), and the mice were placed in cages (6 per cage) in a controlled environment (20–25 °C, 40%–60% relative humidity). Animals were fed a sterile diet (SPF Biotechnology Co., Ltd.), and they could freely access water; each group was implanted in two symmetrical positions (*n* = 10).

Six 18-month-old male beagles (Beijing Fangyuanyuan Animal Farm) were used to establish three diabetic beagles. Each beagle had three loci on one side as the experimental group, so *N* = 9. In addition, three beagles with the same age, sex, weight and normal blood glucose were selected as the negative controls. The process of establishing diabetic dogs was as follows. First, all beagle dogs were fed high-fat and high-calorie feed (5 100 kcal/d: 53% fat, 19% protein, 28% carbohydrate, purchased from Beijing Huabu Biotechnology Company). STZ was injected, and the animals were fasted for 12 h before injection and allowed to drink water freely. Then, intravenous anesthesia with 3% pentobarbital sodium (1 mL/kg, Shanghai National Pharmaceutical Group Chemical Reagent Company) was administered. During the experiment, anesthesia was maintained by intravenous injection of 3% pentobarbital sodium (1 mL/kg). The total dosage of pentobarbital sodium used as a supplement should not exceed 40% of the standard dose (experimental dog weight * 1 mL/kg). After anesthesia, the animal was fixed on the operating table in a back position and disinfected with iodine and 75% alcohol. A sterile sheet was laid out, and 62.5 mg/mL of STZ citric acid solution was administered intravenously to the right saphenous vein of a beagle at a dose of 18.5 mg/kg for 2–3 min. After the injection, 0.9% normal saline was slowly intravenously administered for approximately 30 min. Fasting blood glucose was measured at 2, 4, 6, and 8 weeks after the injection. When the fasting blood glucose of the beagles was 7.0 mmol/L or the blood glucose at 2 h after a meal was >11.1 mmol/L, the diabetes model was successfully established. Then, 0.5 U/kg insulin was subcutaneously injected every other day, and blood glucose was measured 2 h later. When the blood glucose dropped significantly, the blood glucose was stated to have fluctuated. The dynamic changes in blood glucose in dogs within one month are shown in Fig. S6.^50^ Eight weeks before the jawbone defect, the first to fourth premolars on both sides of the beagle’s mandible were surgically extracted. The anesthesia method was the same as before, and attention was given to protecting the cheek, tongue, and lateral walls of the alveolar fossa. Teeth were carefully extracted without damaging the extraction socket. After tooth extraction, 0.1 mg/kg Metacam (PO; Boehringer Ingelheim Co., Ridgefield, CT, USA), 1 mg/kg ketorolac (Toradol 30 mg, Shanghai Roche Pharmaceuticals Co., Ltd., Shanghai, China), 1.7 mg/kg tramadol (Adolonta injectable, Grünenthal, Huayou Medical Group, Beijing, China), and 0.01 mg/kg buprenorphine (Buprex, Reckitt Benckiser Pharmaceuticals Limited, Berkshire, UK) were used to reduce pain. To prevent postoperative infection, we administered amoxicillin (20 mg/kg PO; The United Laboratories Co., Ltd., Hong Kong, China) for 6 days. Each dog was fed a liquid diet for 2 weeks, followed by a soft diet in a single cage. The extraction sites were allowed to heal for 8 weeks.^51^ In the next stage, following the standard procedure, with the same anesthesia and postoperative care methods as previously described, a drilling machine with a diameter and depth of 5 mm was used to drill holes in the mandible of a beagle dog using a minimally invasive method with water cooling. Three cylindrical bone defects with a diameter and depth of 5 mm were made on each side, and an injection was made on the side of the blood glucose fluctuation dog with 100 µl of Exos-Smpd3@Ns. On the other side, 100 µL of PBS was injected as a control, and the wound was sutured layer by layer (Fig. S7).

### Micro-CT evaluation

Prior to mechanical and histological evaluations, a micro-CT system (Siemens, Germany, Invaeon; 80 kV, 500 μ) was used. The specimen was scanned with a 1 500 ms exposure time. The area of interest was selected, mainly a cylinder with a diameter of 5x5x5 mm^3^. The Tb. Th, Tb. N, Tb. Sp, BS/BV, and BV/TV were measured.

### Organizational observation

Nude mouse replanted samples were decalcified in 10% ethylenediaminetetraacetic acid (EDTA; pH 7.4) for 8 weeks and then embedded in paraffin. The slices (5 μm thick) were stained with hematoxylin and eosin (H&E). Images were obtained using an optical microscope (Olympus BX43F, Osaka, Japan) and the accompanying Olympus DP72 digital camera imaging system. The bone defect samples of beagle dogs were fixed with 4% formalin, decalcified with 10% EDTA (pH 7.0), and dehydrated with a series of graded ethanol solutions and 100% acetone. Then, the sample was embedded in methyl methacrylate, and tissue slides (25) were prepared in the buccal lingual direction parallel to the bone defect axis using an EXAKT 400CS grinder (Leica, Wetzlar, Germany). Then, the slides were stained with toluidine blue and Goldner trichrome. Images were captured and analyzed using an optical microscope (Olympus BH2 with S Plan FL2 lens, Tokyo, Japan) and a computerized digital image analysis system (Leica Imaging system, Cambridge, England). Then, Tb. Th, Tb. N, Tb. Sp, BS/BV, and BV/TV were measured.

### Statistical analysis

All statistical calculations were conducted using SPSS 16 statistical software (SPSS, Cary, North Carolina, USA). Statistical significance was determined through independent sample t tests or analysis of variance. A *P*-value less than 0.05 was considered statistically significant: *P < 0.05, **P < 0.01, **P < 0.001, ****P < 0.000 1.

## Supplementary information

西方原始数据

补充信息

## Data Availability

All the data support the figures, and the other findings are available upon reasonable request to the corresponding authors.
