# Supplementary figures and images for: A blood glucose fluctuation-responsive delivery system promotes bone regeneration and the repair function of Smpd3-reprogrammed BMSC-derived exosomes
Source: Int J Oral Sci. 2024 Dec 1;16:65. doi: 10.1038/s41368-024-00328-6 (PMC11608271; doi:10.1038/s41368-024-00328-6)

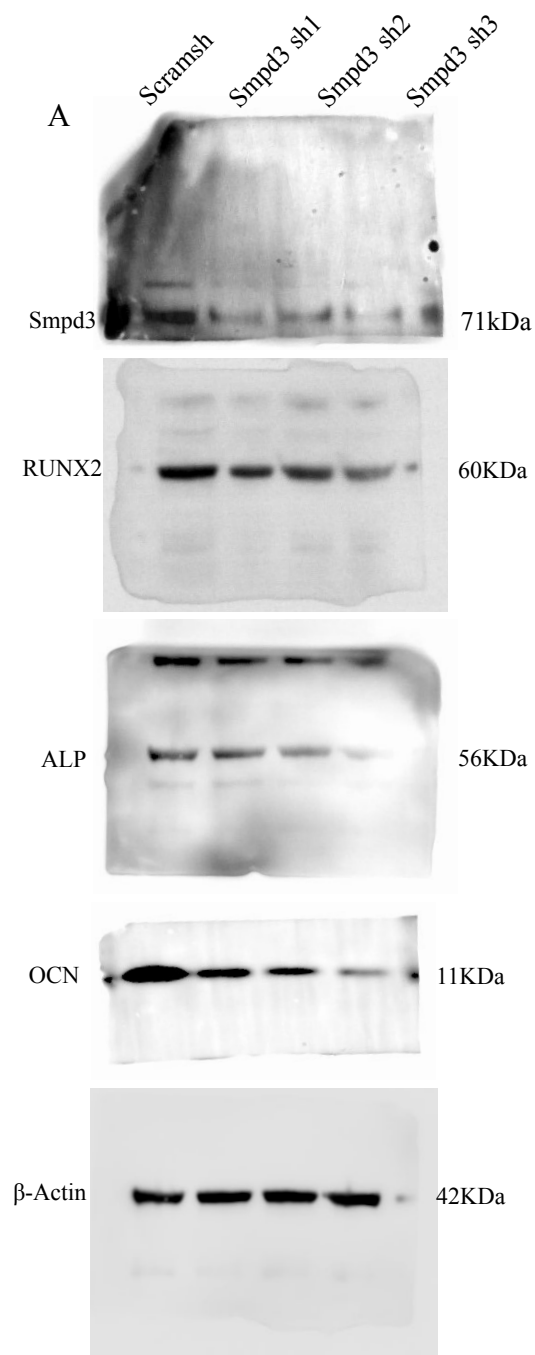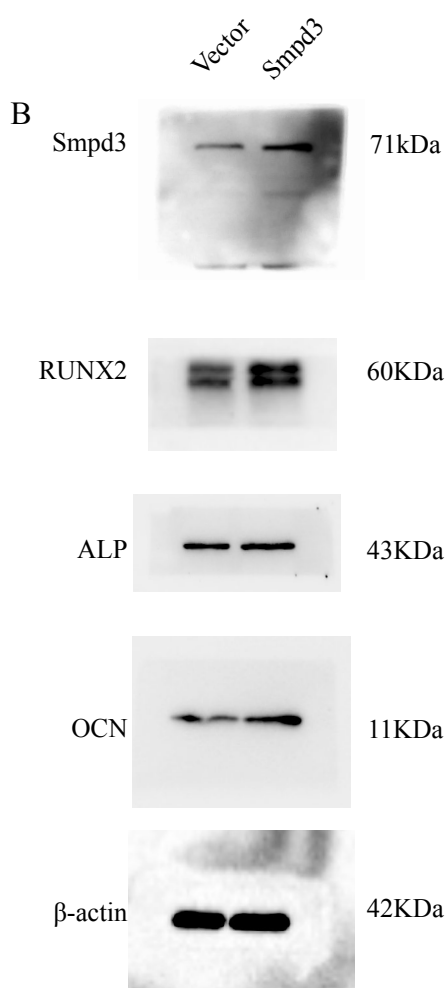

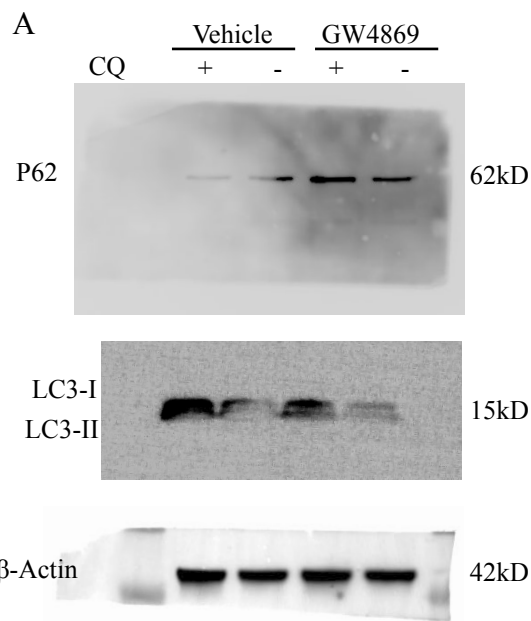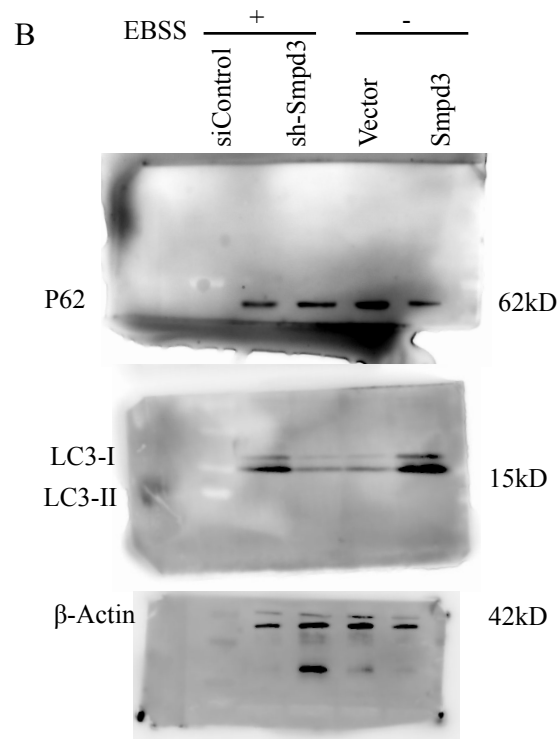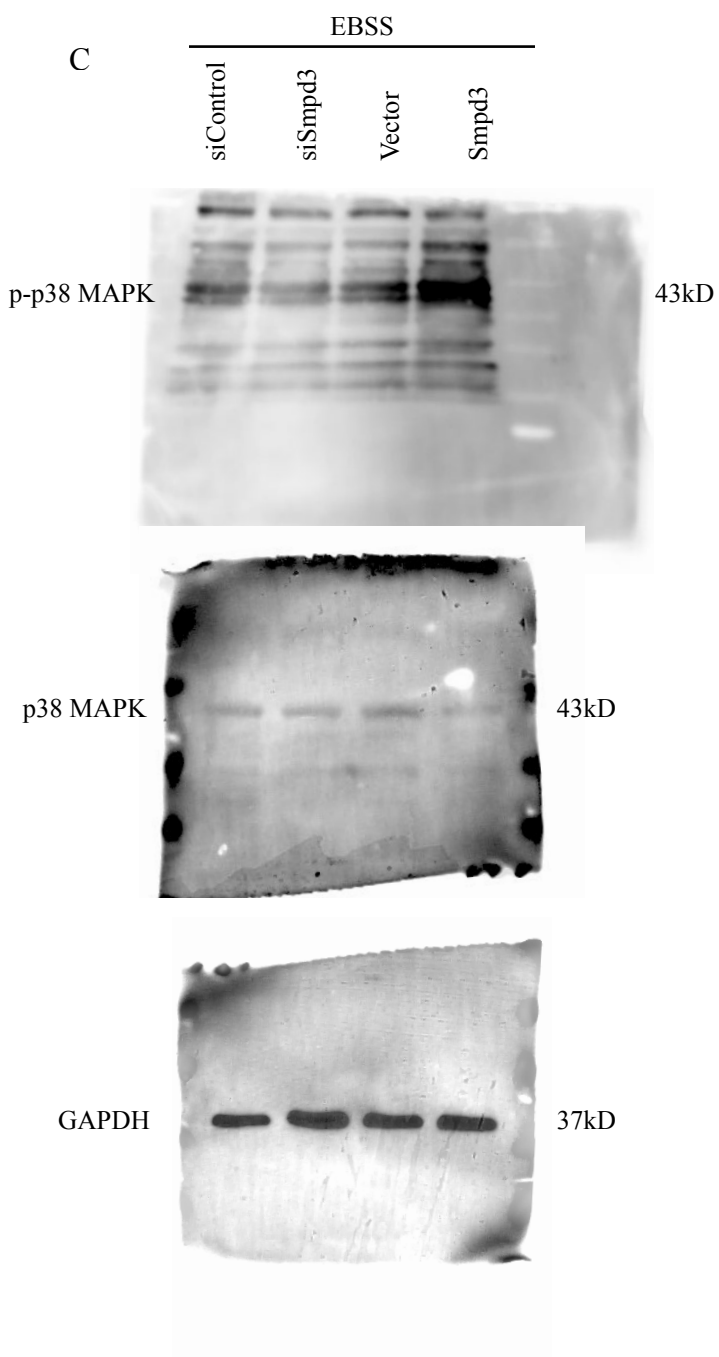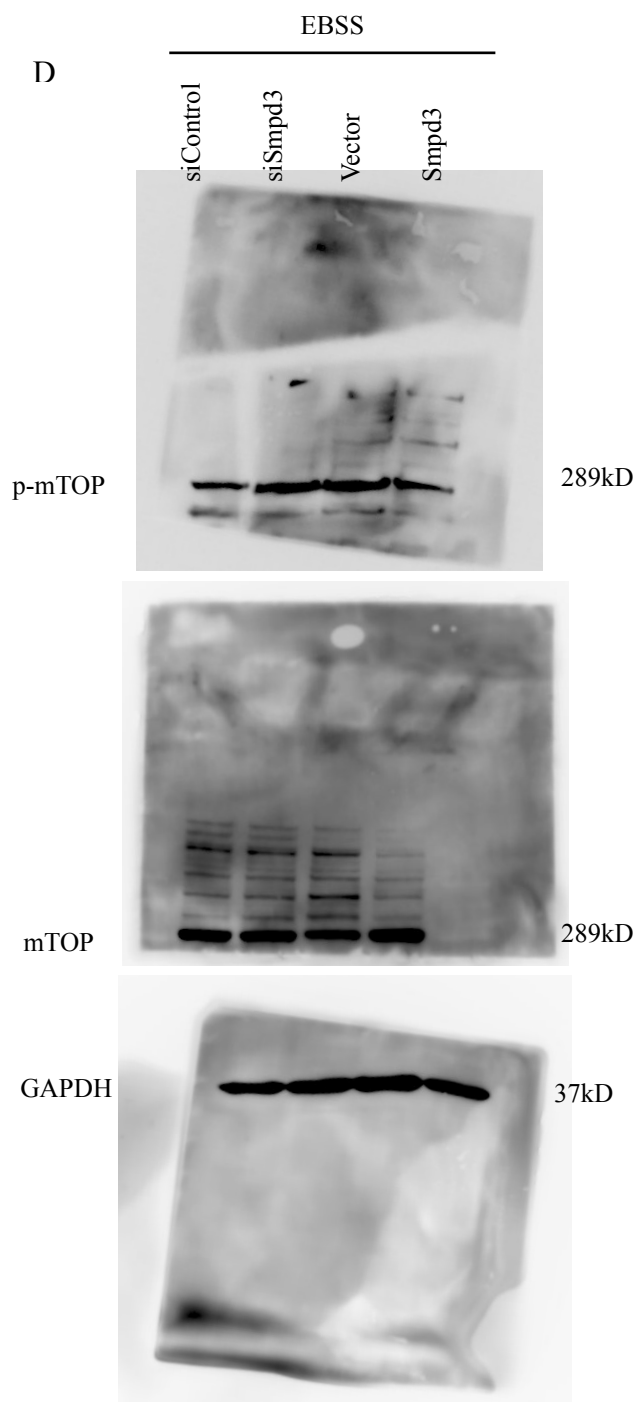

Cells MSC-Eos  
Exos-Smpd3

CD63

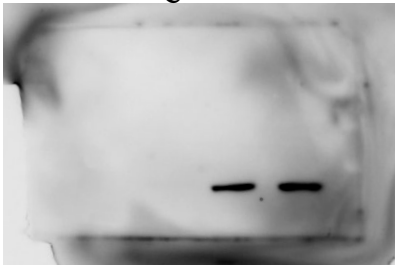

CD9

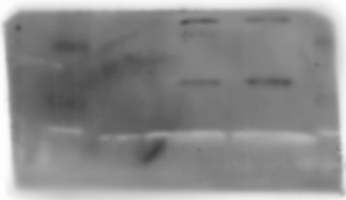

$\beta$ -Tubulin

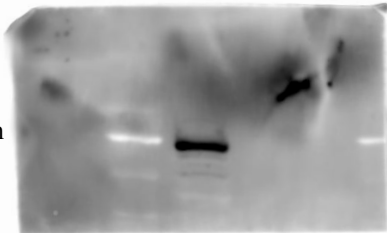

Histon H3

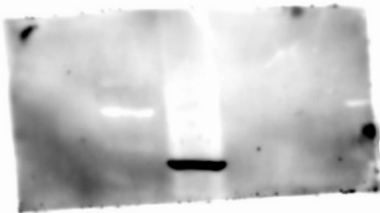

Supplement: Supplementary file 1 — 西方原始数据 [file 41368_2024_328_MOESM1_ESM.pdf]
