# Supplementary material for: A blood glucose fluctuation-responsive delivery system promotes bone regeneration and the repair function of Smpd3-reprogrammed BMSC-derived exosomes
Source: Int J Oral Sci. 2024 Dec 1;16:65. doi: 10.1038/s41368-024-00328-6 (PMC11608271; doi:10.1038/s41368-024-00328-6)
Supplement: Supplementary file 2 — 补充信息 [file 41368_2024_328_MOESM2_ESM.docx]

Supplementary Figure


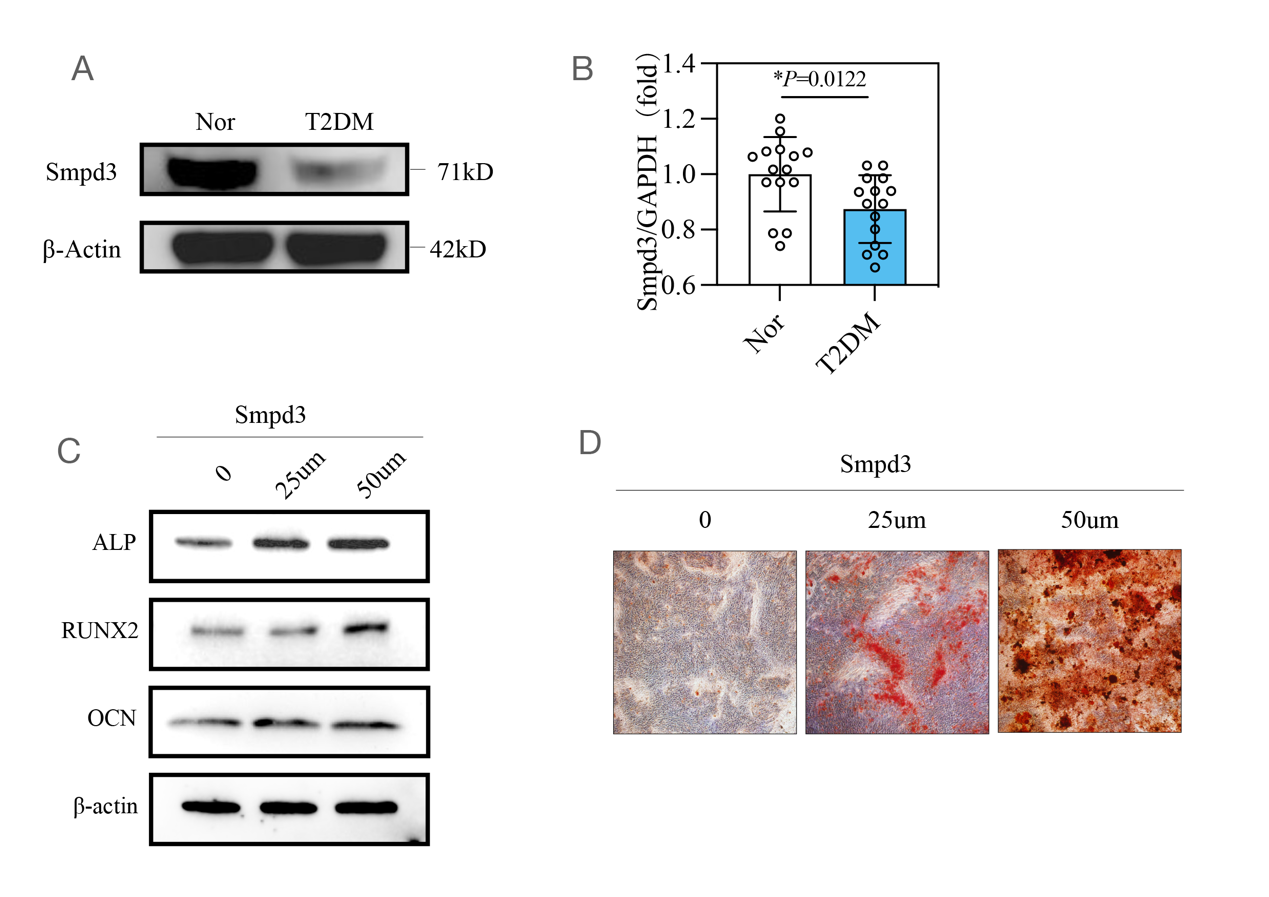


Figure S1. The expression and role of Smpd3 in jawbone-derived BMSCs of patients with T2DM with fluctuating blood glucose. A. At the protein level, Smpd3 expression in jawbone-derived BMSCs from T2DM patients with fluctuating blood glucose decreased B. At the mRNA level, Smpd3 expression in jawbone-derived BMSCs from T2DM patients with fluctuating blood glucose decreased C. With Smpd3 recombinant protein, it was found that 25 µm and 50 µm doses of Smpd3 can promote the expression of the osteogenic markers ALP, RUNX2, and OCN. D. ARS staining revealed that Smpd3 at doses of 25 µm and 50 µm could enhance ARS staining.


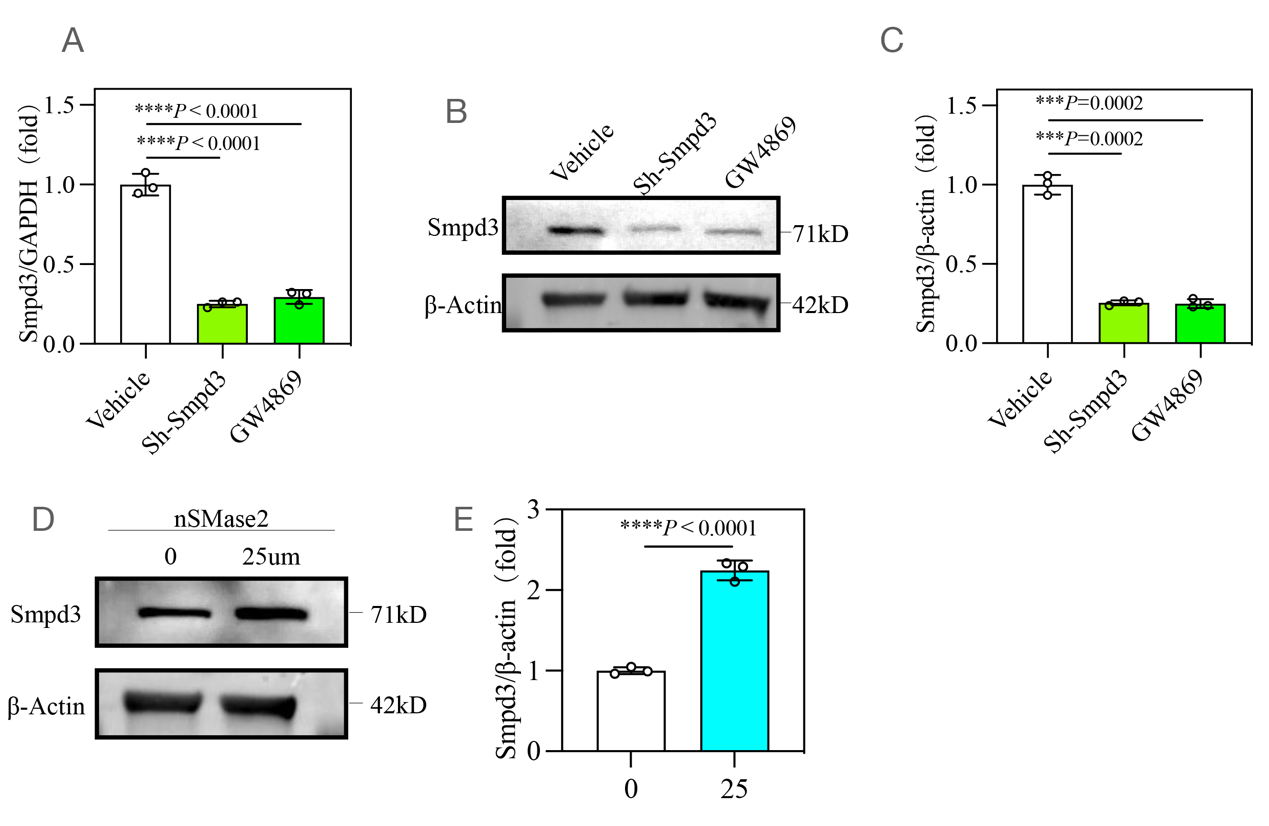


Figure S2. Efficiency of knockdown and overexpression of Smpd3. A. Smpd3 knockdown lentivirus and the Smpd3 inhibitor GW4869 can both inhibit Smpd3 transcription at the mRNA level at the protein level. Smpd3 knockdown lentivirus and the Smpd3 inhibitor GW4869 can both inhibit Smpd3 expression. D and E. Smpd3 can be overexpressed at the protein level using the Smpd3 recombinant protein nSMase2.


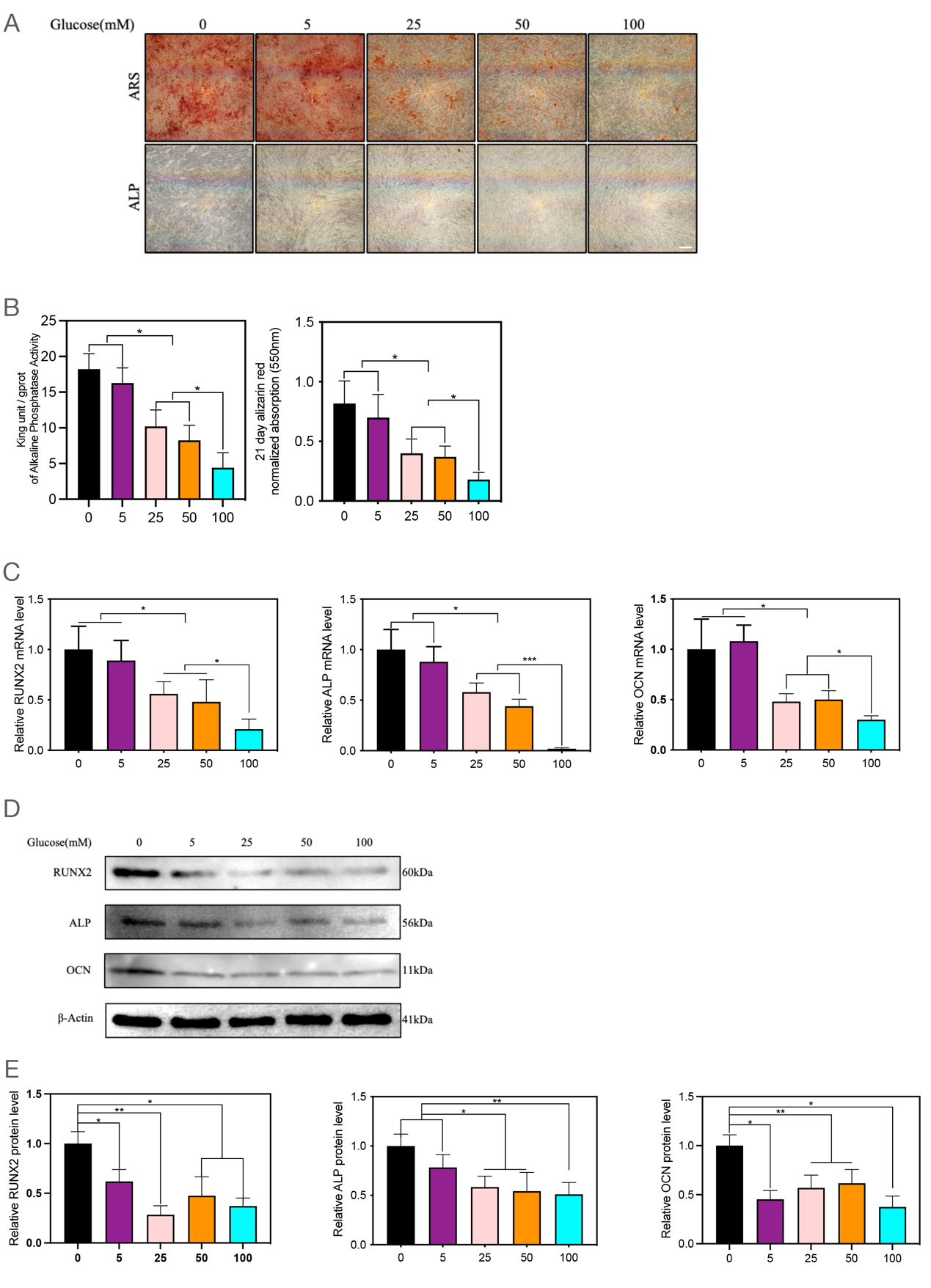


Figure S3. Screening the effects of different concentrations of sugar solutions on the osteogenic differentiation of BMSCs. A. At glucose concentrations of 0/5/25/50/100 mM, 25 mM high glucose had an inhibitory effect on ALP and ARS staining in BMSCs. B. High sugar (25 mM) has an inhibitory effect on ALP activity and ARS quantification. C. At the mRNA level, 25 mM high glucose inhibits the osteogenic markers RUNX2, ALP, and OCN. D and E. At the protein level, 25 mM high sugar inhibits the osteogenic markers RUNX2, ALP, and OCN.


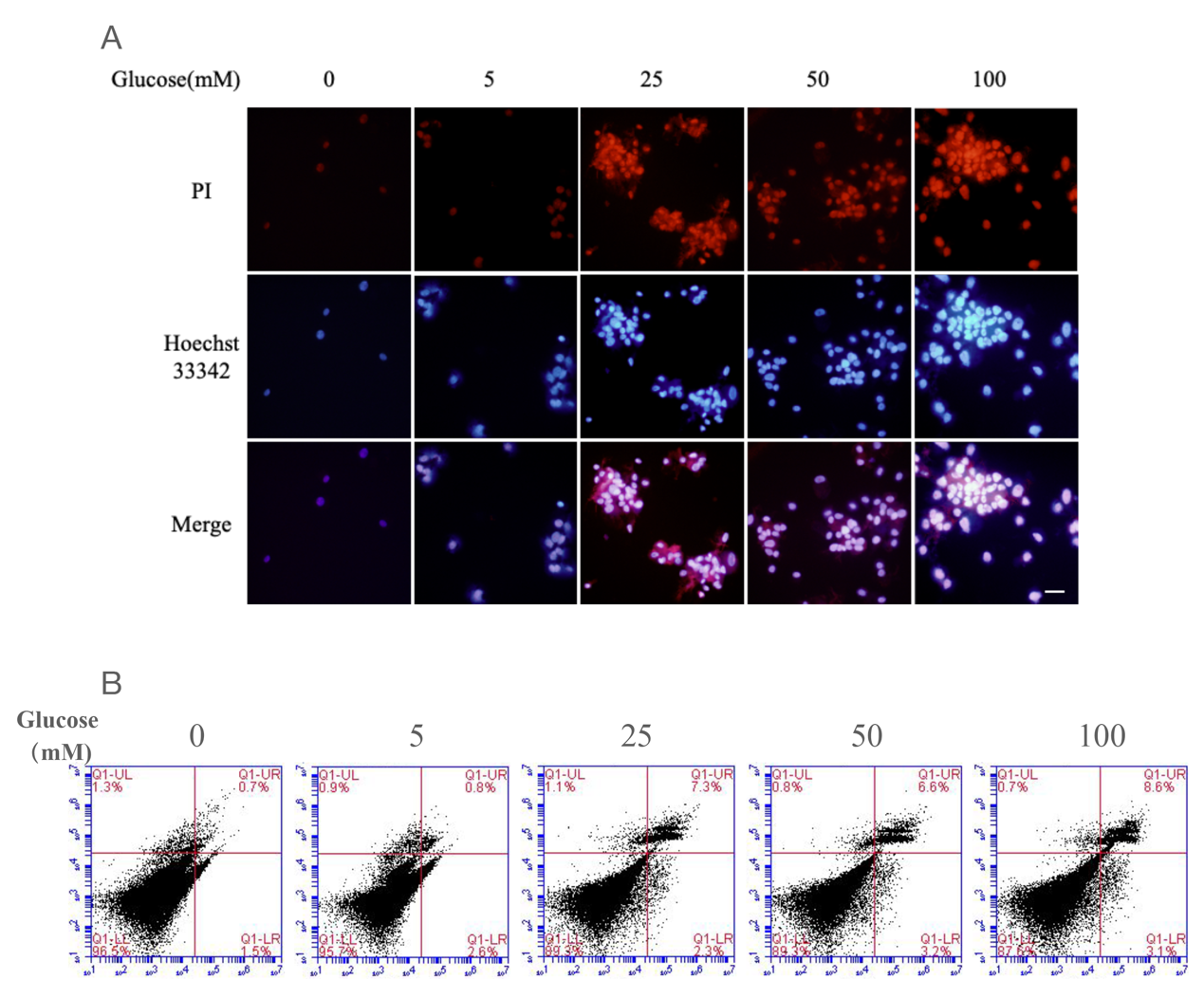


Figure S4. Screening the effects of different concentrations of sugar solutions on BMSC apoptosis. A. The apoptosis detection kit showed that 25 mM high glucose increased PI and Hoechst staining levels. B. Flow cytometry analysis showed that 25 mM high glucose increased early apoptosis levels in BMSCs.
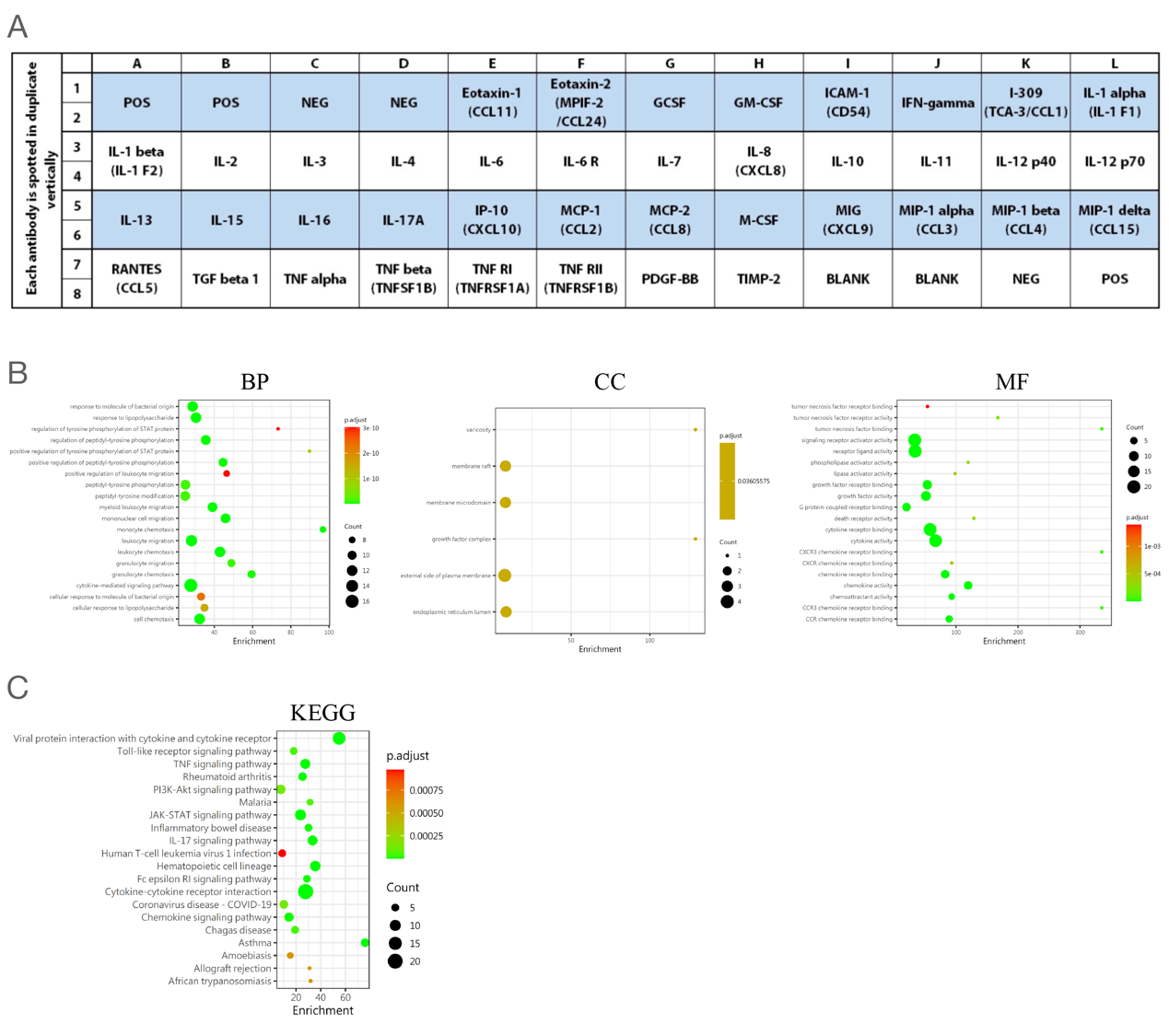


Figure S5. Inflammatory factor detection. A. The names of inflammatory factors corresponding to 41 types of human inflammatory factor membrane chips. B. GO analysis of inflammatory factor detection. C. Analysis of KEGG enrichment pathways for inflammatory factor detection.


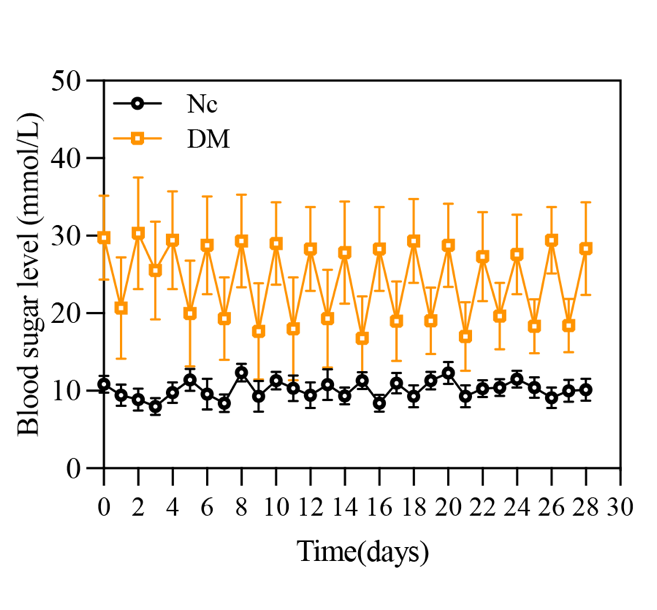


Figure S6. Blood glucose fluctuation. Blood glucose monitoring in diabetic beagles within one month showed that the blood glucose fluctuation range was 16.78 ± 5.43-30.33 ± 7.22 mM/l.


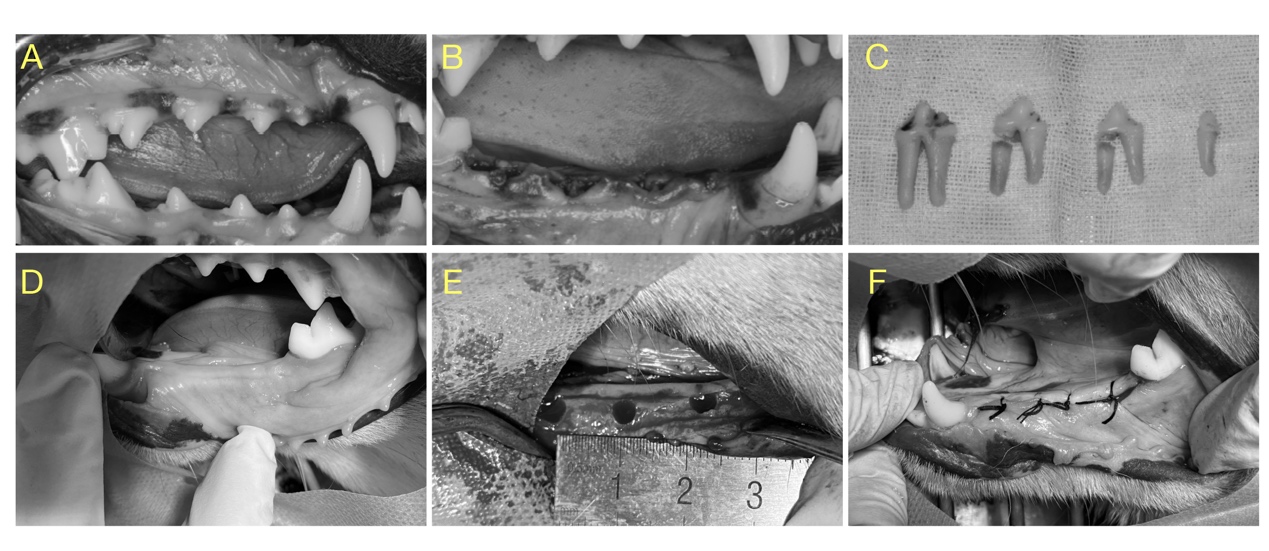


Figure S7. The modeling process of jawbone defects in beagle dogs. A. The integrity of the bicuspid dentition was assessed before tooth extraction. B. The first to fourth premolars were removed from the lower jaw of a beagle. C. The integrity of the tooth root was assessed. D. A surgical area was established for examination before establishing a bone defect. E. Cylindrical bone defects with a diameter of 5 m and a depth of 5 m were prepared and incubated with Exos-Smpd3@Ns. F. The wound was sutured.

Supplementary Table

Table S1. The patients involved in validation queue

| **Patients (T2 DM)** | **Age** | **Gender** | **HbA1c (glycated hemoglobin)** | **Duration of T2 DM (years)** | **Treatment modalities** | **Smoking** | **Patients (Nor)** | **Age** | **Gender** | **Smoking** |
| --- | --- | --- | --- | --- | --- | --- | --- | --- | --- | --- |
| 1 | 46 | Male | 7.7% | 6 | Oral drugs | No | 16 | 47 | Male | No |
| 2 | 57 | Male | 7.5%% | 10 | Oral drugs | No | 17 | 51 | Male | No |
| 3 | 63 | Male | 7.5% | 2 | Diet and exercise | No | 18 | 61 | Male | No |
| 4 | 51 | male | 6.5% | 4 | Insulin injection | No | 19 | 45 | male | No |
| 5 | 72 | Male | 7.6% | 16 | Oral drugs | Yes | 20 | 72 | Male | Yes |
| 6 | 45 | Female | 7.0%-8.0% | 7 | Oral drugs | No | 21 | 45 | Female | No |
| 7 | 59 | Male | 6.0%-7.0% | 6 | Oral drugs | No | 22 | 62 | Male | No |
| 8 | 58 | Male | 6.7% | 1 | Oral drugs | No | 23 | 62 | Male | No |
| 9 | 53 | Male | 6.0%-7.0% | 10 | Insulin injection | No | 24 | 60 | Male | No |
| 10 | 61 | Female | 7.0%-8.0% | 15 | Oral drugs | No | 25 | 60 | Female | No |
| 11 | 62 | Female | 6.0%-7.0% | 20 | Oral drugs | No | 26 | 57 | Female | No |
| 12 | 64 | Female | 6.0%-7.0% | 13 | Oral drugs | No | 27 | 64 | Female | No |
| 13 | 54 | Male | 6.0%-7.0% | 10 | Oral drugs | No | 28 | 58 | Male | No |
| 14 | 62 | Male | 6.0%-7.0% | 10 | Oral drugs | No | 29 | 60 | Male | No |
| 15 | 67 | Male | 6.0%-7.0% | 10 | Insulin injection | No | 30 | 61 | Male | No |

Table S2. Sequences of the primers used for qRT-PCR.

|  | Forward primer (5’ to 3’) | Reverse primer (5’ to 3’) |
| --- | --- | --- |
| Smpd3 | CCTCCTTCATACCCACCACCTAC | AGAGAAAGCCGAGAAACGCAAAG |
| RUNX2 | ACCCATATGTACCATCGATGTC | GAATTCGATGATCAACTCACGG |
| ALP | ATGGGATGGGTGTCTCCACA | CCACGAAGGGGAACTTGTC |
| OCN | CACTCCTCGCCCTATTGGC | CCCTCCTGCTTGGACACAAAG |
| GAPDH | GAAGGTGAAGGTCGGAGTC | GAAGATGGTGATGGGATTTC |
| CD86 | CTGCTCTCTGCTAACTTCAGTCAAC | CACTCATCTTCTTAGGTTCTGGGTAAC |
| iNOS | CTCAGAGTACAGCAAGTGGAAGTTC | GAAAGCAGGAAGCCAGCAGAC |
| CD206 | TCCGACCCTTCCTTGACTAATCC | ATGTCTCCGCTTCATGCCATTG |
| Arg | CTCCTCGTCATCTGTTGTTCCATAC | CTCCTTGTTCTCCACCTGTTTCTTC |
